# Targeting DRD2 by the antipsychotic drug, penfluridol, retards growth of renal cell carcinoma via inducing stemness inhibition and autophagy-mediated apoptosis

**DOI:** 10.1038/s41419-022-04828-3

**Published:** 2022-04-23

**Authors:** Min-Che Tung, Yung-Wei Lin, Wei-Jiunn Lee, Yu-Ching Wen, Yu-Cheng Liu, Ji-Qing Chen, Michael Hsiao, Yi-Chieh Yang, Ming-Hsien Chien

**Affiliations:** 1 Department of Surgery, Tungs’ Taichung MetroHarbor Hospital, Taichung, Taiwan; 2 Graduate Institute of Clinical Medicine, College of Medicine, Taipei Medical University, Taipei, Taiwan; 3 Department of Urology, School of Medicine, College of Medicine and TMU Research Center of Urology and Kidney (TMU-RCUK), Taipei Medical University, Taipei, Taiwan; 4 International Master/PhD Program in Medicine, College of Medicine, Taipei Medical University, Taipei, Taiwan; 5 Department of Medical Education and Research, Wan Fang Hospital, Taipei Medical University, Taipei, Taiwan; 6 Department of Cancer Biology, Geisel School of Medicine at Dartmouth, Lebanon, NH United States; 7 Genomics Research Center, Academia Sinica, Taipei, Taiwan; 8 Department of Medical Research, Tungs’ Taichung MetroHarbor Hospital, Taichung, Taiwan; 9 TMU Research Center of Cancer Translational Medicine, Taipei Medical University, Taipei, Taiwan; 10 Pulmonary Research Center, Wan Fang Hospital, Taipei Medical University, Taipei, Taiwan; 11 Traditional Herbal Medicine Research Center, Taipei Medical University Hospital, Taipei, Taiwan

**Keywords:** Renal cell carcinoma, Cancer stem cells, Oncogenes, Tumour biomarkers

## Abstract

Renal cell carcinoma (RCC) is one of the most lethal genitourinary malignancies with poor prognoses, since it is largely resistant to chemotherapy, radiotherapy, and targeted therapy. The persistence of cancer stem cells (CSCs) is the major cause of treatment failure with RCC. Recent evidence showed that dopamine receptor D2 (DRD2)-targeting antipsychotic drugs such as penfluridol exert oncostatic effects on several cancer types, but the effect of penfluridol on RCC remains unknown. Here, we uncovered penfluridol suppressed in vitro cell growth and in vivo tumorigenicity of various RCC cell lines (Caki-1, 786-O, A498, and ACHN) and enhanced the Sutent (sunitinib)-triggered growth inhibition on clear cell (cc)RCC cell lines. Mechanistically, upregulation of endoplasmic reticulum (ER) stress-induced unfolded protein response (UPR) was critical for autophagy-mediated apoptosis induced by penfluridol. Transcriptional inhibition of OCT4 and Nanog via inhibiting GLI1 was important for penfluridol-induced stemness and proliferation inhibition. The anticancer activities of penfluridol on ccRCC partially occurred through DRD2. In clinical ccRCC specimens, positive correlations of DRD2 with GLI1, OCT4, and Nanog were observed and their expressions were correlated with worse prognoses. Summarizing, DRD2 antagonists such as penfluridol induce UPR signaling and suppress the GLI1/OCT4/Nanog axis in ccRCC cells to reduce their growth through inducing autophagy-mediated apoptosis and stemness inhibition. These drugs can be repurposed as potential agents to treat ccRCC patients.

## Introduction

Renal cell carcinoma (RCC) accounts for almost 90% of kidney tumors and is among the top 10 malignant cancers worldwide. Based on the pathological features, the most common RCC subtype is clear cell (cc)RCC, followed by papillary (p)RCC [1]. Until now, surgical management of primary tumors remains the gold standard of localized RCC treatment. However, around 25%~30% of newly diagnosed patients already have metastatic RCC, and almost 40% of patients that undergo surgical treatment experience RCC recurrence [2]. A high metastatic index of RCC confers resistance to radiation and chemotherapies, and this led to the development of new therapeutic agents such as target-based therapies. For example, the vascular endothelial growth factor (VEGF)-targeted tyrosine kinase inhibitor (TKI), sutent (sunitinib), demonstrated efficacy in treating mRCC. Approximately 70% of patients show an initial response to VEGF TKIs, but patients eventually acquire resistance and the disease progresses as the tumor develops evasive mechanisms in response to VEGF TKI [3, 4]. Therefore, drugs targeting metastasis-initiating cells would be of great interest.

RCC is a very heterogeneous class of tumors, which comprises different populations of tumors that originate from the highly heterogeneous epithelium of renal tubules [5]. Cancer stem cells (CSCs) are small subpopulations of cancer cells harboring the self-renewal ability to drive tumor growth and cause tumor heterogeneity [6]. In addition to cancer development, CSCs are also responsible for disease recurrence, metastasis, and cancer aggressiveness, including treatment resistance such as chemotherapy, radiotherapy, and targeted therapies [4, 7, 8]. Therefore, it would be of great interest to develop new therapeutic drugs that target CSCs, based on molecular mechanisms that regulate stem cell properties.

Dopamine is a major catecholamine neurotransmitter, which binds to dopamine receptors (DRs), and it thus play an important role in the central nervous system (CNS) including sleep, working memory, emotions, learning, and so on. Dysfunction of the dopaminergic system is related to schizophrenia and Parkinson’s disease (PD), and targeting DRs by antagonists is the principal approach [9]. In addition to their roles in the CNS, the dopamine-DR axis also has peripheral functions in regulating gastrointestinal motility, cardiovascular function, immune system, and kidney function [10–12]. DRs are G protein-coupled receptors comprising DRD1, DRD2, DRD3, DRD4, and DRD5, which are divided into D1-like receptors (DRD1 and DRD5) and D2-like receptors (DRD2, DRD3, and DRD4) according to their different regulatory actions against adenylyl cyclase (AC) activity [10]. Previous epidemiological studies declared that patients with schizophrenia or PD who receive DR antagonists have a lower cancer incidence [13–15], suggesting that DR antagonists may be suitable for repurposing for cancer treatment. Actually, increasing evidence shows that DRs, especially DRD2, is associated with the regulation of tumor behaviors in various cancer types, including proliferation, apoptosis, autophagy, and stemness [16]. For example, pimozide, a DRD2 inhibitor, was reported to induce cell-cycle G_1_ arrest and apoptosis in pancreatic cancer cells [17]. Another DRD2 blocker, thioridazine, was shown to induce autophagy in ovarian cancer cells [18]. Trifluoperazine, a clinically used antipsychotic that targets DRD2, was demonstrated to suppress growth of CSCs and overcome drug resistance of lung cancer [19].

Penfluridol, a diphenylbutylpiperidine antipsychotic drug, has a longer elimination half-life than thioridazine, trifluoperazine, and pimozide when used to treat schizophrenia via targeting DRD2 [20, 21]. Recently, a few studies including those from our laboratory investigated the anticancer effects of penfluridol on several cancer types, especially breast, lung, colon, pancreatic, and brain cancers [20, 22, 23]. Until now, the anticancer potential of penfluridol against RCC is still unknown. Moreover, none of the previous studies investigated the anticancer activity of penfluridol that is due to D2-receptor antagonism. Therefore, this study investigated the anticancer effects of penfluridol against RCC in vitro and in a tumor xenograft model and examined possible underlying mechanisms including DRD2. We further investigated whether penfluridol treatment could potentially suppress RCC tumor growth through the eradication of RCC CSCs.

## Materials and Methods

### Materials

Penfluridol (P3371), dopamine hydrochloride (H8502), quinpirole hydrochloride (Q102), 4-phenylbutyric acid (4-PBA), 3-methylamphetamine (3-MA), chloroquine (CQ), and their solvent, dimethyl sulfoxide (DMSO), were purchased from Sigma-Aldrich (St. Louis, MO, USA). GLI1 inhibitor, GANT 58 (14193) was purchased from Cayman Chemical (Ann Arbor, MI, USA). SAR405 and Bafilomycin A were respectively purchased from MedChemExpress (Monmouth Junction, NJ, USA) and ChemCruz (TE, Huissen, Netherland). Primary antibodies against cleaved caspase-3, cleaved poly(ADP ribose)polymerase (PARP), LC3, p62, and unfolded protein response (UPR) pathways including glucose-related protein 78 (GRP78), CCAAT-enhancer-binding protein homolog protein (CHOP), and phosphorylated (p)-eIF2α were purchased from Cell Signaling Technology (Danvers, MA, USA). Antibodies specific for Beclin-1, GLI1, OCT4, lamin A/C, and Nanog were obtained from Santa Cruz Biotechnology (Santa Cruz, CA, USA) or Abcam (Cambridge, UK). GAPDH and α-tubulin loading control antibodies were purchased from Proteintech (Rosemont, IL, USA). The OCT4 CR4 pGreenFire Response Reporter was purchased from System Biosciences (Palo Alto, CA, USA). Fetal bovine serum (FBS), antibiotics, and all medium additives were obtained from Life Technologies (Gaithersburg, MD, USA). Unless otherwise specified, other chemicals used in this study were purchased from Sigma Chemical (St. Louis, MO, USA).

### Cell lines and cell culture

The 786-O, Caki-1, A498, and ACHN RCC cell lines with different Von Hippel-Lindau (VHL) statuses were obtained from the American Type Culture Collection (ATCC; Manassas, VA, USA). Cells were maintained in RPMI-1640 (for 786-O) or minimum essential medium (MEM; for Caki-1, ACHN, and A498) media supplemented with 10% FBS, 2 mmol/L L-glutamine, 100 U/mL penicillin, and 100 μg/mL streptomycin in a humidified incubator containing 5% CO_2_ at 37 °C. All cells were routinely tested and were negative for mycoplasma.

### Cell viability assay

The cytotoxic effects of penfluridol and sunitinib on cell viability were determined by a 3-(4,5-dimethylthiazol-2-yl)-2,5-diphenyltetrazolium bromide (MTT) or CCK-8 (Sigma-Aldrich) assay. Cells were seeded at a density of 10^4^ cells/well in 96-well plates and incubated for 24 h. Then RCC cells were treated with indicated concentrations of penfluridol for another 24 h. At the end of the incubation period, the medium was removed, and 200 μl of MTT was added or 10 μl of WST-8 was directly added without removing the medium. Viable cells reduce MTT or WST-8 to a colored formazan product, and this was detected at 570 or 490 nm absorbance using a microplate reader (MQX200; Bio-Tek Instruments, Winooski, VT, USA).

### Colony-forming assay

RCC cells (10^3^ cells/well) were seeded into six-well dishes for 24 h of incubation and further treated with penfluridol at the indicated concentration (0.25~5 μM) for another 24 h. Cells were then continuously incubated in new fresh medium with or without penfluridol for 7~10 days, cells were fixed with methanol and stained with 0.1% crystal violet, and colonies were manually counted using free ImageJ software (National Institutes of Health, Bethesda, MD, USA).

### Sphere-forming assay

RCC cells (5 × 10^3^ cells/well) were seeded in ultra-low attachment six-well plates (Corning, Tewksbury, MA, USA) containing serum-free medium supplemented with 2% B-27 supplement (ThermoFisher Scientific, Rockford, IL, USA), 20 ng/ml recombinant human epidermal growth factor (EGF) and fibroblast growth factor (FGF; Corning), and penicillin/streptomycin. After 7~10 days of incubation at 37 °C in a 5% CO_2_ humidified atmosphere, each sphere was treated with penfluridol when its diameter reached 100 μm. After the penfluridol (2.5 µM) treatment for 72 h, the number of spheroid colonies was measured under an inverted phase-contrast microscope.

### Lentiviral production and infection

293 T packaging cells were co-transfected with 10 μg of the OCT4 CR4 pGreenFire Response Reporter construct or (sh)RNA construct of DRD2 (target sequence: GCC CTT CTT CAT CAC ACA CAT) together with 10 μg of pCMVDR8.91 (the packaging vector) and 1 μg of pMD.G (the envelope vector). The transfection medium was removed and replaced with fresh culture medium after 16 h of incubation. At 48 h later, viral supernatants were collected through centrifugation at 1500 rpm for 5 min. Then, RCC cells were infected with fresh lentivirus-containing medium (supplemented with 8 μg/mL polybrene) for 24 h and subjected to the following assays.

### Extraction of RNA and reverse-transcription quantitative polymerase chain reaction (RT-qPCR)

Total RNA was extracted from RCC cells using the TRIzol reagent (Invitrogen Life Technologies, Carlsbad, CA, USA) and reverse-transcribed into complementary (c)DNA using an iScript™ cDNA Synthesis kit (Bio-Rad). Gene amplification was carried out using OmicsGreen 5X qPCR Master Mix with StepOnePlus™ Real-Time PCR Systems (Applied Biosystems, Carlsbad, CA, USA). GAPDH was used to normalize messenger (m)RNA levels, and primer sequences involved in the RT-qPCR are listed in Table 1. Gene expressions were normalized using ΔCt relative to GAPDH. Expression changes were analyzed with the 2^−ΔΔCt^ method [24].

**Table 1:** The primer sequences used in RT-qPCR analysis.

Primer Name	Sequence
OCT4_F	CTTGCTGCAGAAGTGGGTGGAGGAA
OCT4_R	CTGCAGTGTGGGTTTCGGGCA
Nanog_F	AATACCTCAGCCTCCAGCAGATG
Nanog_R	TGCGTCACACCATTGCTATTCTTC
GAPDH_F	GTCCACTGGCGTCTTCACCACC
GAPDH_R	AGGCATTGCTGATGATCTTGAGGC

### Tumorigenicity in vivo xenograft and limiting dilution assay

Caki-1 cells were treated with vehicle or 5 μM penfluridol for 48 h and resuspended at 10^4^, 10^5^, or 10^6^ cells in 100 μl PBS. Suspended cells were mixed with 100 μl Matrigel (BD Biosciences, San Jose, CA, USA) and subcutaneously injected into the right flank of NOD-scid IL2Rγ^null^ (NSG) mice (4~5 weeks old). After transplantation, the tumor size was measured weekly with calipers, and the tumor volume was estimated by the following formula: tumor volume (mm^3^) = 1/2 × length × width^2^. At the end of the experiment, tumors were dissected out, and immunohistochemical (IHC) staining was performed. The experimental protocols used herein were evaluated and approved by the Academia Sinica Institutional Animal Care and Utilization Committee (19-11-1367).

### Supravital cell staining with acridine orange (AO) to detect autophagy

Basic evidence of autophagy induced in cells is the gradual formation of acidic vesicular organelles (AVOs) [25]. Herein, RCC cells were stained with AO (Sigma) to detect the formation and accumulation of AVOs according to previously published procedures [26]. Briefly, cells were seeded into 24-well plates and treated with 7.5 μM penfluridol for 18 h. Subsequently, AO was added to the medium at a final concentration of 1 μg/mL for 15 min, and cells were then washed with phosphate-buffered saline (PBS). In AO-stained cells, the cytoplasm and nuclei fluoresce bright green, whereas acidic compartments fluoresce bright red. Images were captured with a Zeiss Axiophot fluorescence microscope (Carl Zeiss Microimaging, Gottingen, Germany).

### Nuclear and cytosolic protein extraction and Western blot analysis

For whole-cell protein extraction, cells were washed with cold PBS and lysed with radioimmunoprecipitation assay (RIPA) buffer as described previously [27]. For nuclear and cytosolic protein extraction, protein extracts were prepared using an NE-PER Cytoplasmic and Nuclear Protein extraction kit according to the manufacturer’s instructions (ThermoFisher Scientific, Rockford, IL, USA). The protein concentration was determined with a Bio-Rad protein assay reagent (Bio-Rad, Hercules, CA, USA), with bovine serum albumin (BSA) as the standard. A Western blot analysis was performed with indicated primary antibodies and horseradish peroxidase-conjugated secondary antibodies according to previously described detailed processes [27].

### Morphological assessment of apoptotic cells by Hoechst 33342 staining

To identify morphological changes in cells under apoptosis, 2.5 × 10^5^ RCC cells were seeded in 6-cm dishes and incubated at 37 °C until cells had grown to about 70~80% confluency. Cells were then treated with vehicle or 7.5 µM penfluridol for another 24 h, the medium was replaced by fresh medium, and Hoechst 33342 (1:1000) was added at 37 °C for 30 min. Nuclear morphological changes were examined and photographed with a Zeiss Axiophot fluorescence microscope.

### Luciferase reporter assay

RCC cells (2.5 × 10^5^) bearing the OCT4-CR4 response reporter were seeded in 6-cm dishes at 37 °C for 24 h and treated with vehicle or penfluridol for another 24 h. Cell lysates were then harvested, and firefly luciferase activity was determined using a luciferase assay kit (Promega, Madison, WI, USA) with a SpectraMax M2 luminescence microplate reader (Molecular Devices, San Jose, CA, USA). The relative luciferase activity was calculated according to the formula: relative luciferase activity (fold) = (luciferase activity of sample treated with penfluridol) / (luciferase activity of sample treated with vehicle).

### IHC staining

All tumor tissue samples were processed as described in our previous study [22]. PBS was used to wash the slides before antibody incubation. The GLI1 or Ki67 antibody was then applied to slides at a dilution of 1:100, incubated at 4 °C overnight, and washed twice with PBS. Next, slides were developed with a VECTASTAIN ABC (avidin-biotin complex) peroxidase kit (Vector Laboratories, Burlingame, CA, USA) and a 3,3’-diaminobenzidine (DAB) peroxidase substrate kit (Vector Laboratories) according to the manufacturer’s instructions. Nuclei were counterstained with hematoxylin.

### Bioinformatics analysis

Clinical analysis of molecular expression using RNA sequencing in a cohort of patients with ccRCC was obtained from The Cancer Genome Atlas (TCGA) University of California Santa Cruz Xena website (https://xenabrowser.net/). In addition, the Gene Expression Omnibus (GEO) database was used for the analysis of *DRD2* mRNA levels in normal and ccRCC tissues. The 216938_x_at DRD2 probe was used for the GSE15641 analysis. The prognostic significance of *OCT4*, *Nanog*, *GLI1*, and *DRD2* levels or the combined effects of these genes in patients with ccRCC were determined using a Kaplan-Meier analysis. Correlations of *GLI1* with *OCT4* or *Nanog*, and correlations of *DRD2* with *GLI1*, *OCT4*, *Nanog*, *EGFR*, or *PRKACA* in ccRCC were evaluated using the cBioportal platform (https://www.cbioportal.org/).

### Statistical analysis

Values are presented as the mean ± standard deviation (SD). Statistical analyses were performed using SPSS v20 (SPSS, Chicago, IL, USA), and quantified data were analyzed using GraphPad Prism 7 (GraphPad Software, San Diego, CA, USA). Differences between two groups were analyzed using Student’s *t*-test and were considered significant at *p* < 0.05.

## Results

### Penfluridol attenuates the proliferative and colony-forming abilities of human RCC cells harboring different VHL statuses and enhances the antiproliferative effect of sunitinib

**Fig. 1:**
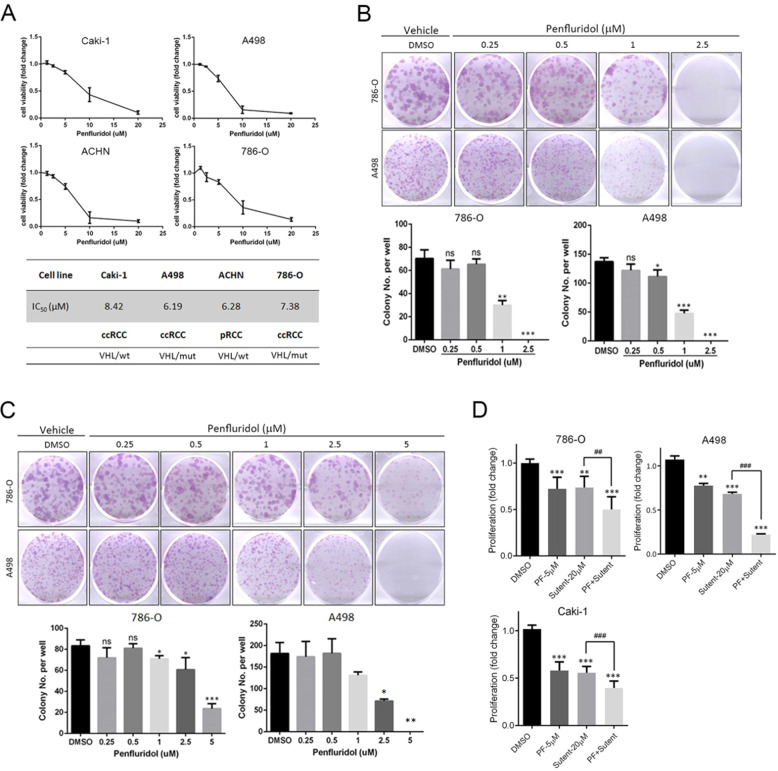
Short-term and long-term proliferation inhibitory effects of penfluridol on renal cell carcinoma (RCC). **A** Four RCC cell lines including papillary type (ACHN) and clear cell type (Caki-1, A498, and 786-O), were treated with the indicated concentrations of penfluridol (1.25, 2.5, 5, 10 and 20 μM) or dimethyl sulfoxide (vehicle control) for 24 h, and an MTT assay was performed to determine cell viability. Data are presented as the mean ± standard deviation (SD) from three independent experiments. Values of the half maximal inhibitory concentration (IC_50_) of these cells are shown in the lower panel. **B** and **C** Colony-forming abilities of 786-O and A498 cells after treating cells with the indicated concentrations of penfluridol for 7 days (**B**) or 24 h (**C**). Upper panels of **B**, **C**: representative photomicrographs. Lower panels of **B**, **C**: values are presented as the mean ± SD from three independent experiments. *p < 0.05, **p < 0.01, and ***p < 0.001 compared to those of the vehicle group. ns: not significant. **D** After treatment of A498, 786-O, or Caki-1 cells with 5 μM penfluridol and/or 20 μM sunitinib/sutent for 24 h, the proliferative abilities of these ccRCC cells were determined by a CCK-8 assay. **p < 0.01 and ***p < 0.001 compared to those of the vehicle group. ^##^*p* < 0.01 and ^###^*p* < 0.001 compared to those of the sunitinib/sutent only group.

ccRCC represents 75% of all RCC cases, and about 80% of ccRCC cases harbor mutations of the tumour-suppressive gene, *VHL* [28]. Herein, we used four RCC cell lines including ccRCC (Caki-1, A498, and 786-O) and pRCC (ACHN) harboring either the wild-type (WT) or mutant *VHL* gene to evaluate short-term treatment (24 h) effects of penfluridol at the indicated concentrations (0, 1.25, 2.5, 5, 10 and 20 μM) on cell proliferation. According to results from the MTT assay, values of the half-maximal inhibitory concentration (IC_50_) of penfluridol on cell viability were 8.42, 6.19, 7.38, and 6.28 μM in Caki-1, A498, 786-O, and ACHN cells, respectively (Fig. 1A). In addition, a colony formation assay was used to evaluate the long-term anticancer effect of penfluridol. After treating ccRCC cells (A498, and 786-O) with penfluridol for 7 days, the colony-forming abilities of those cells were all concentration-dependently suppressed by penfluridol, and penfluridol at 1 μM showed significant anti-colony-forming activity (Fig. 1B). Even though 24 h treatment with 2.5 μM penfluridol still effectively reduced the colony-formation ability of 786-O and A498 cells (Fig. 1C), we further observed the enhanced effects of penfluridol in combination with sunitinib/sutent, first-line therapy for RCC, on inhibition of proliferation of ccRCC cells (Fig. 1D). These results suggested that penfluridol would likely be useful as a therapeutic agent in managing ccRCC.

### Penfluridol triggers autophagic cell death in ccRCC cells via inducing the ER stress-mediated UPR

**Fig. 2:**
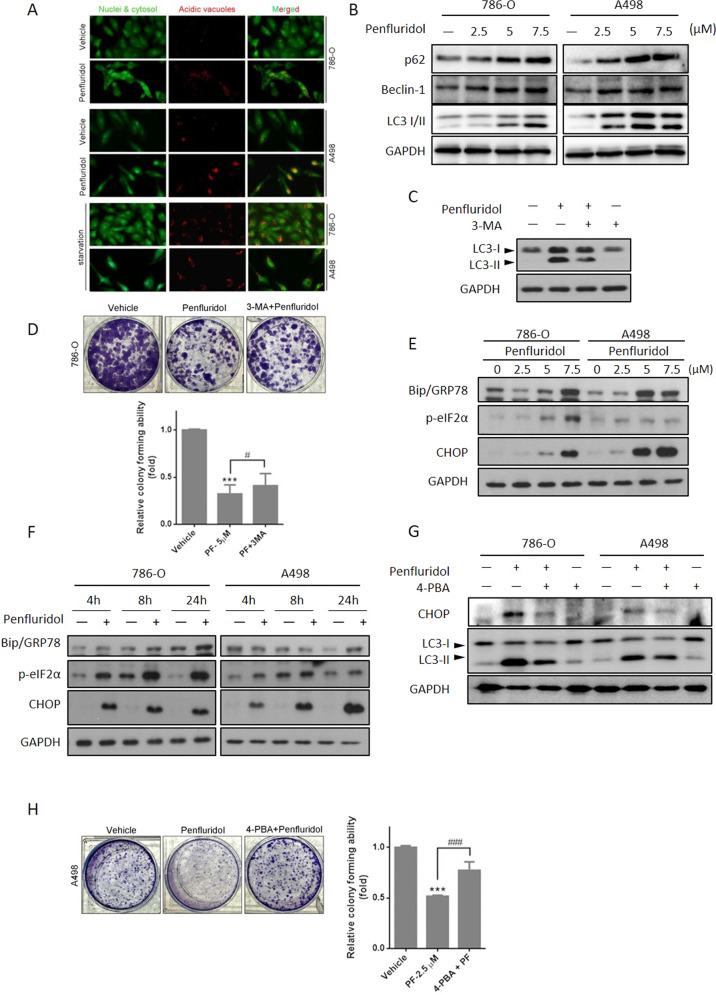
Autophagic cell death triggered by penfluridol via inducing the endoplasmic reticular (ER) stress-mediated unfolded protein response (UPR). **A** Upper panel: Acridine orange staining was used to detect acidic vesicular organelle (AVO) formation after 7.5 μM penfluridol treatment for 18 h in A498 and 786-O cells and analysis under a fluorescence microscope. Lower panel: The same cells were serum-starved for 48 h as a positive control of cell autophagy. **B** A498 and 786-O cells were treated with various concentrations of penfluridol for 18 and 24 h, respectively and the expression of p62 and Beclin-1, and turnover of LC3 were detected by a Western blot (WB) analysis. **C** and **D** 786-O cells were pretreated with 20 μM of 3-methylamphetamine (3-MA) for 1 h, followed by penfluridol treatment for 24 h. LC3 turnover and growth inhibitory effects triggered by penfluridol and penfluridol + 3-MA were respectively determined by a WB analysis (**C)** and colony-formation assay (**D**). **E** and **F** A498 and 786-O cells were treated with various concentrations of penfluridol for 24 h (**E**) or 7.5 μM penfluridol for the indicated time points (**F**), and unfolded protein response (UPR) signals, glucose-related protein 78 (GRP78), CCAAT-enhancer-binding protein homologous protein (CHOP), and phosphorylated eIF2α were detected by a WB analysis. **G** 786-O and A498 cells were pretreated with or without 4-phenylbutyric acid (4-PBA) (1 mM) for 1 h, followed by penfluridol (7.5 μM) treatment for 24 h. LC3 turnover and CHOP expression in both cells were detected by a WB analysis. **H** A498 cells were treated with either penfluridol or penfluridol + 4-PBA, and the colony-forming abilities were further evaluated. *** *p* < 0.001 compared to those of the vehicle group. ^#^ *p* < 0.05 and ^###^ *p* < 0.001 compared to those of the penfluridol only group.

To further elucidate the type of cell death caused by penfluridol, ccRCC cells treated with penfluridol were tested for autophagy. Autophagy is characterized by AVO formation, which is detected by vital staining with AO, and we observed that the number of AVOs was significantly higher in penfluridol-treated 786-O and A498 cells compared to the untreated group (Fig. 2A, upper panel). Serum-starvation of both ccRCC cell lines was used as a positive control for cells undergoing autophagy (Fig. 2A, lower panel). Levels of LC3-II (an autophagosome component) were positively correlated with autophagy, and we observed that penfluridol treatment induced concentration-dependent conversion of LC3 I to LC3 II in ccRCC cell lines. Other autophagy markers including p62 (an adaptor protein for fusion autophagosome and autolysosome degradation) and Beclin-1 (critical for phagophore membrane nucleation) were also induced by penfluridol in ccRCC cell lines (Fig. 2B and Supplementary Fig. 1). In addition, we observed that the autophagy inhibitors, 3-MA and SAR405, reversed penfluridol-induced turnover of LC3 or inhibition of colony formation and proliferation in ccRCC cells (Fig. 2C, D and Supplementary Fig. 2), suggesting that penfluridol may confer cytotoxicity partly through inducing autophagic cell death. Previous studies demonstrated that misfolded proteins accumulating in the ER can trigger the ER stress-mediated UPR and further induce autophagic cell death in cancer cells [29]. We next investigated whether penfluridol induced ER stress in ccRCC cells. UPR-related chaperone proteins and other UPR-related markers in penfluridol-treated ccRCC cells (786-O and A498) were evaluated by a Western blot analysis which showed that penfluridol treatment caused concentration (2.5, 5, and 7.5 μM)- and time-dependent (4, 8, and 24 h) induction of glucose-related protein 78 (GRP78) and CCAAT-enhancer-binding protein homologous protein (CHOP) expressions, and phosphorylation (p) of eIF2α (Fig. 2E and F). To further investigate the linkage between penfluridol-induced ER stress and autophagy in ccRCC cells, the ER stress inhibitors 4-phenylbutyric acid (4-PBA) was used. We found that penfluridol-induced increases in CHOP and LC3-II were all significantly reversed by both ER stress inhibitors in 786-O, A498, and Caki-1 cells (Fig. 2G and Supplementary Fig. 3). Pretreatment with 4-PBA also significantly reversed penfluridol-induced inhibition of colony formation in A498 cells (Fig. 2H). Taken together, the ER stress-mediated UPR is involved in penfluridol-induced autophagic cell death in ccRCC cells.

### Coordinated regulation of autophagy and apoptosis is involved in ER stress-mediated cytotoxic effects of penfluridol in ccRCC cells

**Fig. 3:**
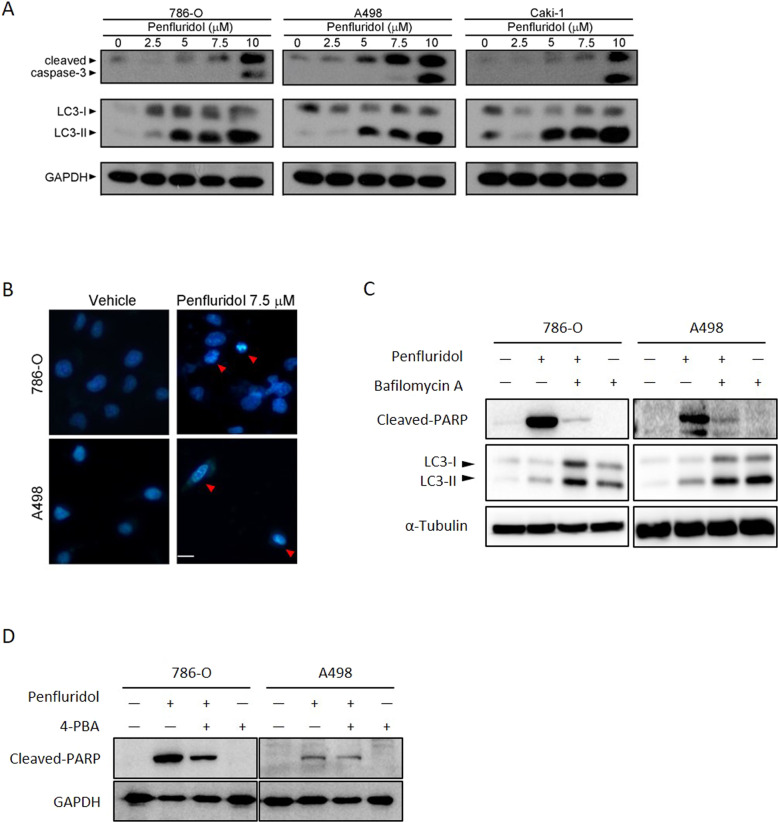
Coordinated regulation of autophagy and apoptosis is involved in the death-inducing effects of penfluridol in clear cell renal cell carcinoma (ccRCC) cells. **A** 786-O, A498, and Caki-1 cells were treated with the indicated concentrations of penfluridol for 24 h. Cell lysates were probed with antibodies against cleaved caspase-3 and LC3. **B** A498 and 786-O cells were treated with 7.5 μM penfluridol for 24 h, and nuclear fragmentation and condensation (red arrows) as indicators of apoptosis were analyzed by fluorescence microscopy after Hoechst 33342 staining (scale bar: 50 μm). **C** A498 and 786-O cells were pretreated with 20 nM of Bafilomycin A for 1 h followed by penfluridol (7.5 μM) treatment for 24 h. Expression levels of LC3 and cleaved poly(ADP ribose) polymerase (PARP) were detected by a Western blot (WB) analysis. **D** 786-O (left panel) and A498 (right panel) cells were pretreated with or without 4-phenylbutyric acid (4-PBA) (1 mM) for 1 h, followed by penfluridol (7.5 μM) treatment for 24 h. Cleaved PARP in both cells were detected by a WB analysis. GAPDH or α-Tubulin were used as an equal loading control.

Autophagy and apoptosis have taken center stage as the principal mechanisms of programmed cell death in mammalian tissues [30]. Moreover, CHOP plays an important role in ER stress-induced apoptosis [31]. Therefore, we further checked if apoptosis also plays a role in the death of ccRCC cells induced by penfluridol. Caspase-3 activation-mediated PARP cleavage is a hallmark of apoptosis. Results from Western blotting showed that penfluridol concentration-dependently induced LC3-II formation and caspase-3 activation in 786-O, A498, and Caki-1 ccRCC cells, and the dominant inducing effects of LC3 turnover and caspase-3 cleavage were respectively observed at 5 and 7.5 μM of penfluridol treatment (Fig. 3A). A similar cytotoxic effect of penfluridol was observed in the ACHN pRCC cell line (Supplementary Fig. 4). Moreover, according to data from the staining of 7.5 µM penfluridol-treated ccRCC cells with Hoechst 33342, we found typical morphological changes of cellular apoptosis, such as condensed nuclear chromatin and nuclear fragmentation (Fig. 3B), suggesting that penfluridol can also induce apoptotic cell death in RCC. To further investigate the crosstalk between ER stress-mediated autophagy and apoptosis induced by penfluridol, the early autophagy inhibitor, 3-MA, and the late autophagy inhibitor, Bafilomycin A and CQ, were used. Pretreatment of RCC cells with Bafilomycin A or CQ respectively enhanced penfluridol-induced LC3-II production and rescued penfluridol-induced PARP cleavage, compared to that of penfluridol treatment only (Fig. 3C and Supplementary Fig. 5, Left panel). The penfluridol-induced apoptotic effect was also reversed by the early autophagy inhibitor, 3-MA (Supplementary Fig. 5, Right panel). In addition, pretreatment of 786-O and A498 cells with 4-PBA, significantly reversed the penfluridol-induced increase of PARP cleavage (Fig. 3D). These results indicated that RCC cell growth inhibition modulated by penfluridol was associated with induction of ER stress-triggered autophagy leading to apoptosis.

### Penfluridol attenuates the self-renewal potential of ccRCC cells through regulating pluripotent transcription factors

**Fig. 4:**
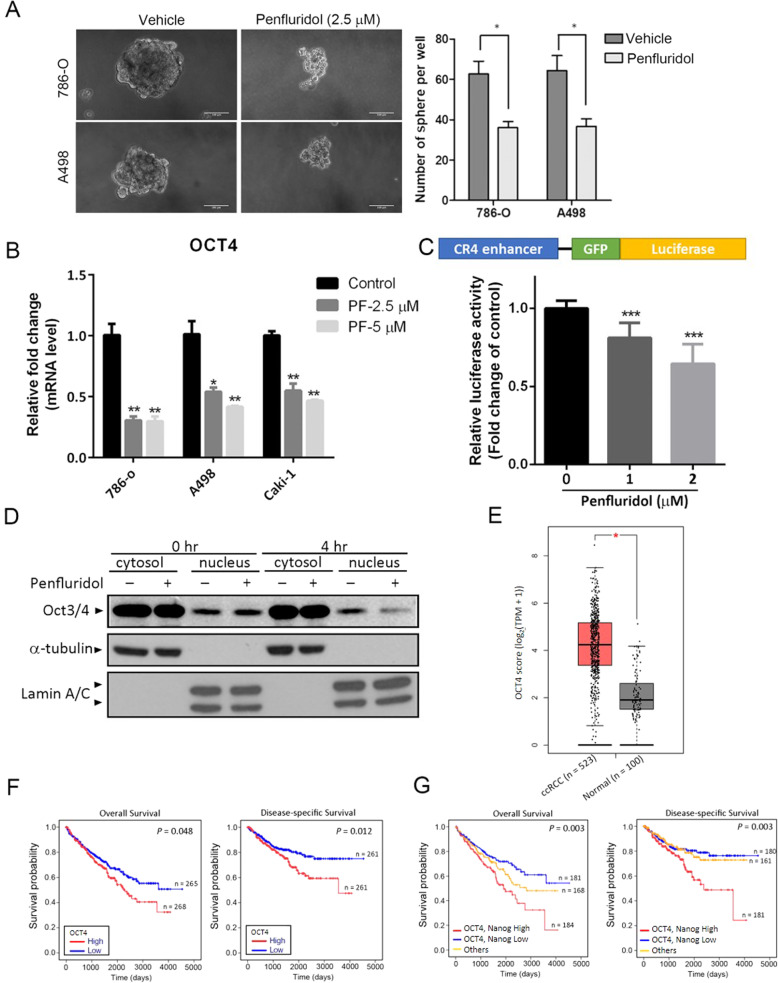
Penfluridol inhibits spherogenicity of clear cell renal cell carcinoma (ccRCC) cells through targeting pluripotent transcription factors. **A** 786-O and A498 cells were seeded on ultra-low attachment six-well plates which allowed for tumor spheroid formation. Several days after seeding, spheroids had formed (with an approximate size of 100 μm), and then they were treated with 2.5 µM of penfluridol for 72 h. Representative images of spheroids derived from both cell lines are shown in the left panels. Scare bar, 100 μm. Spheroids numbers are reported in the right panels. *p < 0.05 compared to those of the vehicle group. **B** ccRCC cells were treated with vehicle or penfluridol at indicated concentrations for 72 h to detect mRNA levels of OCT4 using an RT-qPCR. Quantitative results of OCT4 mRNA levels were adjusted to GAPDH mRNA levels. **C** 786-O cells were transfected with the CR4 region of the OCT4 promoter-driven luciferase construct for 24 h and then treated with the vehicle or penfluridol at indicated concentrations. After 24 h of treatment, luciferase activities were further determined. Values from **B** and **C** are presented as the mean ± standard deviation (SD) of three independent experiments. *p < 0.05, **p < 0.01, and ***p < 0.001 compared to those of the vehicle group. **D** A498 cells were treated with 5 μM penfluridol or the vehicle for 4 h. Cells were harvested and fractionated into cytoplasmic and nuclear fractions and then subjected to a Western blot analysis to detect OCT4 levels. α-Tubulin and lamin A/C were respectively used as the cytosolic and nuclear loading controls. **E** Gene Expression Profiling Interactive Analysis (GEPIA) was performed to validate higher expression of OCT4 transcript in ccRCC samples compared to normal samples. The red box is the cancer tissue group (*n* = 523), and gray is the normal tissue group (*n* = 100). *p < 0.05 compared to the normal tissue grou*p*. **F** Kaplan-Meier plot of overall (left panel) and disease-specific (right panel) survival of patients with ccRCC stratified by OCT4 expression levels. A log-rank test was used to show differences between the groups. **G** Combined analysis of the influence of OCT4 and Nanog expressions on the overall (left panel) and disease-specific (right panel) survival rates of ccRCC patients. The ccRCC dataset from **F** and **G** was retrieved from TCGA.

Previous studies demonstrated that penfluridol can attenuate CSCs of glioblastoma multiforme (GBM) [32]. Sphere formation is regarded as a defining trait of CSCs, so we next investigated whether penfluridol could suppress the volume of ccRCC spheroids using a sphere-forming assay. From photomicrographs presented in Fig. 4A, we observed that both 786-O and A498 ccRCC cells could grow as spheroids in 3D culture. Compared to the vehicle-treated group, 786-O or A498 spheroids treated with 2.5 µM penfluridol for 72 h exhibited decreased sphere diameters (left panel) and numbers (right panel). Taken together, our results suggested that penfluridol not only induced cell death of ccRCC cells, but also reduced the growth of ccRCC spheroids. Given the inhibitory effect of penfluridol on the CSC phenotype, its effect on pluripotent transcription factors such as OCT4, which endows a self-renewal property of cancer cells [33], was then evaluated in ccRCC cells. As shown in Fig. 4B, after treatment of 786-O, A498, and Caki-1 cells with penfluridol for 72 h, mRNA expression levels of OCT4 had significant decreased. OCT4 conserved region 4 (CR4)-driven luciferase reporter activity was also suppressed by receiving penfluridol in 786-O cells in a concentration-dependent manner (Fig. 4C). In addition to the transcriptional inhibitory effect of penfluridol on the *OCT4* gene, nuclear localization of the OCT4 protein was also blocked after 4 h of treatment of A498 ccRCC cells with penfluridol (Fig. 4D). In addition to OCT4, the mRNA expression and nuclear localization of Nanog, a downstream target of OCT4 [34], were also suppressed by penfluridol treatment in ccRCC cells (Supplementary Fig. 6). In clinical specimens, significantly higher OCT4 transcripts were observed in ccRCC tumors compared to normal tissues from TCGA and Genotype-Tissue Expression (GTEx) datasets (Fig. 4E). The Kaplan-Meier (KM) plot revealed a shorter overall survival (OS) and disease-specific survival (DSS) of ccRCC patients with high OCT4 or Nanog expression than patients with low OCT4 or Nanog expression (Fig. 4F and Supplementary Fig. 7). Moreover, from the same TCGA database described above, patients with OCT4^high^/Nanog^high^ ccRCC tumors had the worst OS and DSS rates compared to those with OCT4^high^/Nanog^low^, OCT4^low^/Nanog^high^, or OCT4^low^/Nanog^low^ tumors (Fig. 4G). Taken together, clinical data indicated that the upregulation of OCT4 and Nanog is a critical event in promoting the progression of ccRCC. Negative regulation of CSC properties in ccRCC by penfluridol might be through targeting OCT4 and Nanog.

### Penfluridol suppresses growth of ccRCC via targeting GLI1-mediated expressions of pluripotent transcription factors

**Fig. 5:**
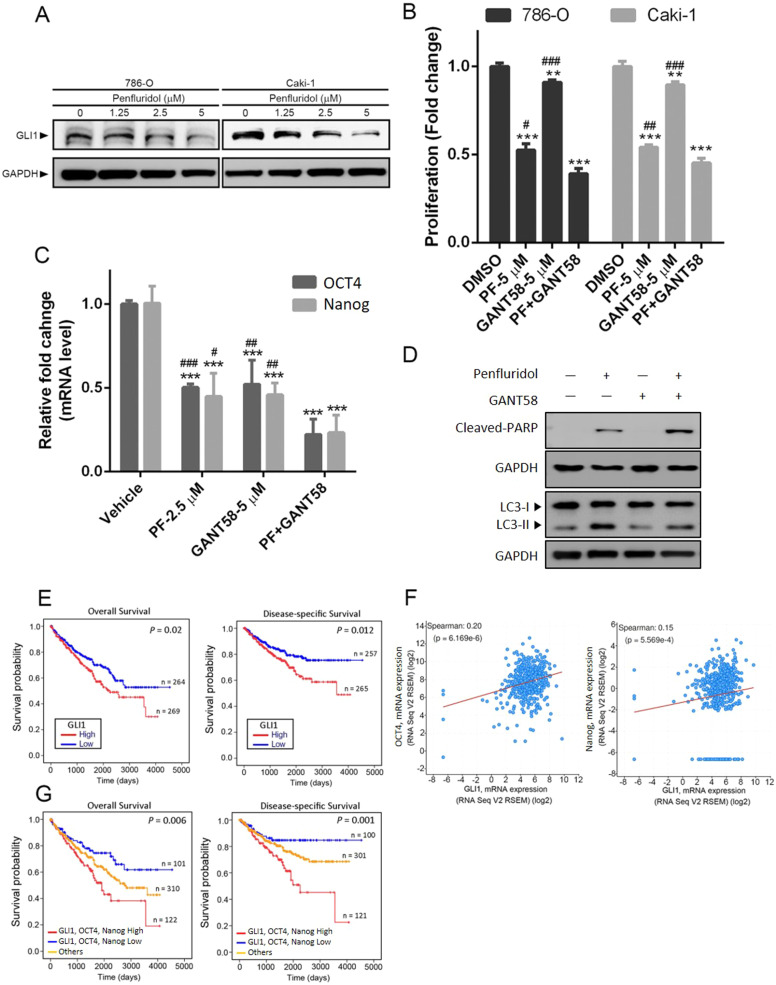
Penfluridol inhibits growth of clear cell renal cell carcinoma (ccRCC) cells via targeting GLI1-mediated expressions of pluripotent transcription factors. **A** 786-O and Caki-1 cells were treated with the indicated concentrations of penfluridol for 24 h. Cell lysates were probed with antibodies against GLI1 and GAPDH. **B** and **C** 786-O or Caki-1 cells were pretreated with GANT58 (5 μM) and penfluridol treatment for 24 or 48 h. The cell proliferative ability and mRNA levels of pluripotent transcription factors (OCT4 and Nanog) were respectively measured by CCK8 (**B**) and RT-qPCR (**C**) analyses. Quantitative results of OCT4 and Nanog mRNA levels were adjusted to GAPDH mRNA levels. **p < 0.01 and ***p < 0.001 compared to the vehicle group. ^#^*p* < 0.05, ^##^*p* < 0.01, and ^###^*p* < 0.001 compared to the penfluridol+GANT58 group. **D** 786-O cells were pretreated with GANT58 (5 μM) for 1 h followed by penfluridol (7.5 μM) treatment for 24 h. LC3 turnover and poly(ADP ribose) polymerase (PARP) cleavage in cells were detected by a Western blot analysis. GAPDH was used as an equal loading control. **E** Kaplan-Meier plot of overall (left panel) and disease-specific (right panel) survival of patients with ccRCC stratified by GLI1 expression levels. A log-rank test was used to show differences between groups. **F** Correlation analysis of The Cancer Genome Atlas (TCGA) ccRCC databases (TCGA, PanCancer Atlas) using cBioPortal revealed the positive correlation between GLI1 expression and OCT4 or Nanog. **G** Combined analysis of the influence of GLI1, OCT4, and Nanog expressions on the overall (left panel) and disease-specific (right panel) survival rates of ccRCC patients. The ccRCC dataset from **E** and **G** was retrieved from TCGA.

GLI1, an effector molecule of the Hedgehog (Hh) pathway, was reported to promote CSC properties by inducing expressions of OCT4, Nanog, and Sox2 [32]. We further tested whether penfluridol can suppress the OCT4-mediated growth of ccRCC via targeting GLI1. A Western blot analysis identified decreased expression of GLI1 following penfluridol treatment in ccRCC cells (786-O, Caki-1, and A498) harboring the WT or mutant *VHL* gene (Fig. 5A and Supplementary Fig. 8). Next, we investigated relationships among penfluridol-mediated inhibition of GLI1, stemness, and cell growth. Treatment of ccRCC cells with the GLI1-specific inhibitor, GANT58, significantly inhibited cell proliferation (Fig. 5B) and OCT4 and Nanog mRNA expressions (Fig. 5C). Combined treatment with GANT58 and penfluridol further enhanced penfluridol-induced inhibition of cell proliferation, OCT4 and Nanog mRNA expressions, and induction of PARP cleavage, but not LC3 turnover (Fig. 5B–D). Taken together, these results indicated that GLI1-mediated expressions of OCT4 and Nanog might play critical roles in anti-CSC efficacy of penfluridol against ccRCC growth. Clinical evidence showed that high GLI1 expression was correlated with poor OS and DSS in patients with ccRCC (Fig. 5E). We further analyzed 510 ccRCC human samples using the cBioportal platform and observed that GLI1 expression was significantly correlated with OCT4 or Nanog expression (Fig. 5F). Moreover, patients with GLI1^high^/ OCT4^high^/Nanog^high^ ccRCC tumors had the shortest OS and DSS times compared to the other groups (Fig. 5G).

### Suppression of tumorigenesis by penfluridol in human ccRCC xenograft models

**Fig. 6:**
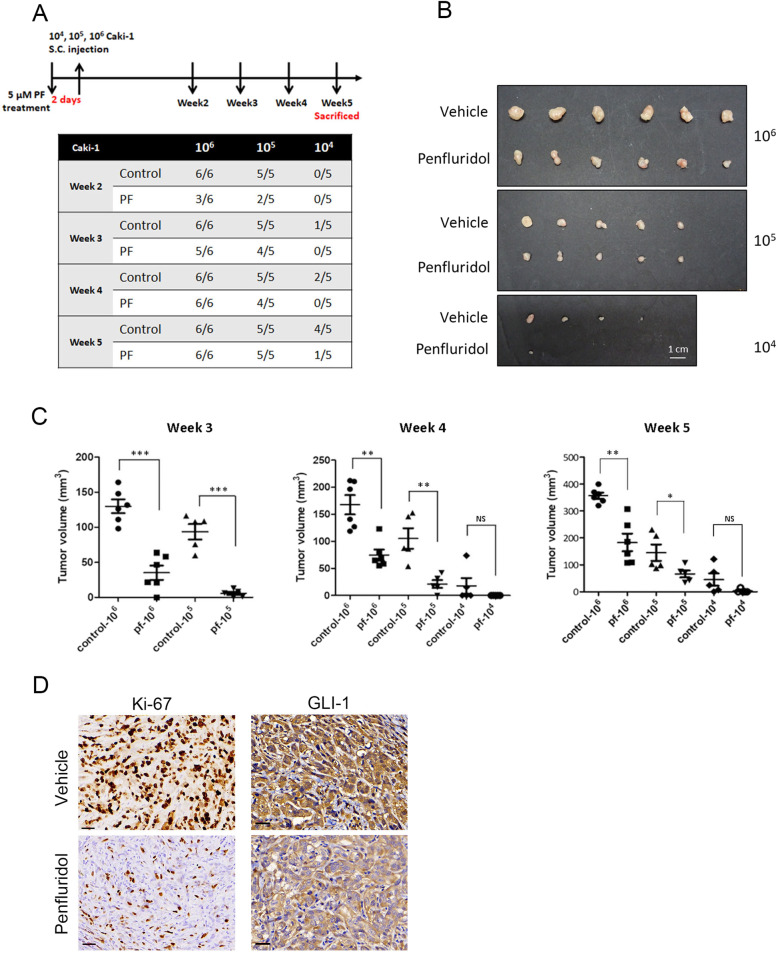
Tumorigenicity in vivo xenograft and limiting dilution assay. **A** Upper panel, timeline of the in vivo study design for investigating the antitumor activity of penfluridol. Caki-1 clear cell renal cell carcinoma (ccRCC) cells that had been treated with penfluridol (5 µM) or the vehicle. For the limiting dilution analyses, the indicated numbers of cells were subcutaneously injected into male NOD-scid IL2Rγ^null^ (NSG) mice (*n* = 5 or 6 per group). Two weeks after tumor cell injection, the tumor volume in mice was checked every week. Lower panel, numbers of tumors generated and results of the limiting dilution assay. Numbers of tumors generated by the cell lines were recorded every week and are shown in the table. **B** All mice were sacrificed at 5 weeks after tumor cell injection, and tumors were dissected and photographed. **C** Tumor development from vehicle- and penfluridol-treated groups. Tumor volumes were recorded at the 3rd, 4th, and 5th weeks after tumor cell injection and are reported as the mean ± standard error of the mean (SEM). *p < 0.05, **p < 0.01, and ***p < 0.001 compared to the vehicle group. NS, not significant. **D** Caki-1 xenografts treated with vehicle or penfluridol were isolated to detect expressions of Ki67 and GLI1 by IHC staining. Original magnification, 400x. Scale bar, 30 μm.

Since CSCs are defined by their higher tumor-initiating ability, we subsequently determined if the anti-CSC activity of penfluridol occurs in vivo using xenotransplantation limiting dilution assays (LDAs). Vehicle- and penfluridol-pretreated Caki-1 cells were subcutaneously injected into NSG mice with serially diluted cell numbers. Two weeks after cells were injected, vehicle-treated Caki-1 cells initiated tumor formation in six of six mice, five of five mice, and none of five mice injected with 10^6^, 10^5^, and 10^4^ cells, respectively. In contrast to vehicle-treated Caki-1 cells, penfluridol-treated cells initiated tumor formation in three of six mice, two of five mice, and none of five mice injected with 10^6^, 10^5^, and 10^4^ cells, respectively. After 5 weeks after injection of 10^4^ cells, tumor formation was observed in four of five mice and one of five mice in vehicle- and penfluridol-treated cells, respectively (Fig. 6A). Although xenografts injected with 10^6^ or 10^5^ cells all produced tumors by the 5th week, tumor sizes in the penfluridol-treated groups were markedly reduced compared to those of the vehicle-treated groups (Fig. 6B). Similar results were observed in tumor volumes recorded at the 3rd, 4th, and 5th weeks after tumor cell inoculation (Fig. 6C), indicating that tumors derived from penfluridol-treated Caki-1 cells grew more slowly than did tumors derived from vehicle-treated Caki-1 cells. After terminating the experiment, tumors were removed and analyzed by IHC. In agreement with our in vitro findings, we observed downregulation of GLI1 and proliferation index Ki-67 in penfluridol-treated tumors compared to vehicle-treated tumors. These results indicated that penfluridol suppresses tumorigenesis of ccRCC by suppressing GLI1-driven tumor growth.

### Penfluridol induces ER stress-mediated cell death and stemness inhibition through targeting DRD2 in ccRCC cells

**Fig. 7:**
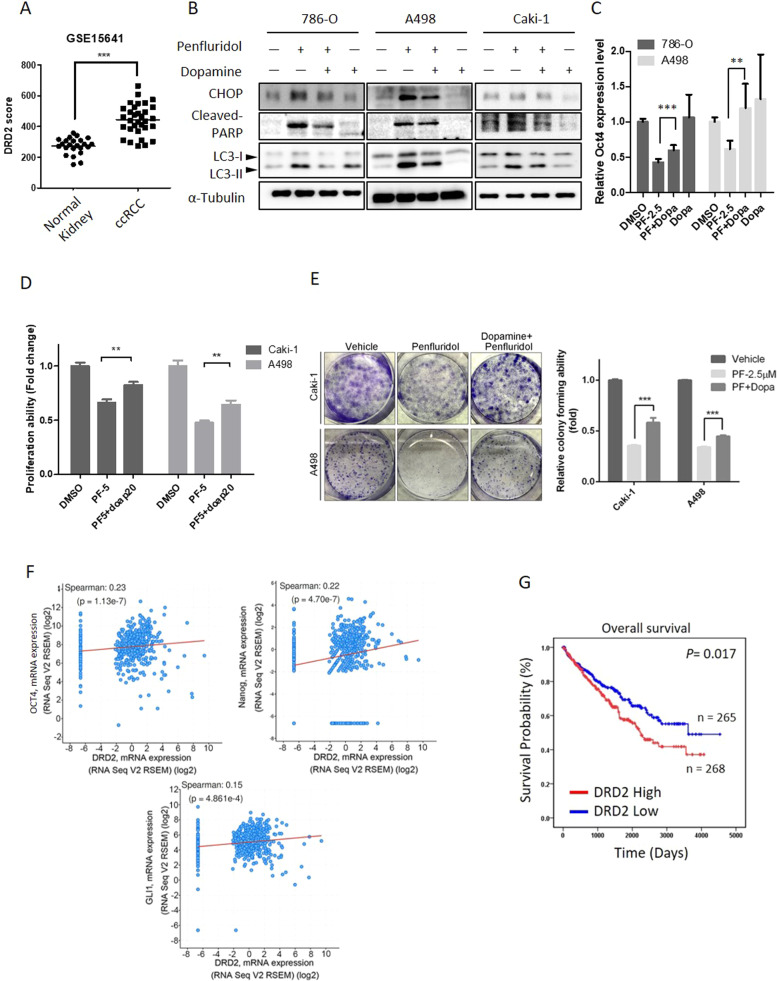
Dopamine receptor D2 (DRD2) is targeted by penfluridol to inhibit growth of clear cell renal cell carcinoma (ccRCC) cells. **A** Gene expression of DRD2 in normal kidney and ccRCC tumor tissues (GSE15641). **B** 786-O, A498 and Caki-1 cells were pretreated with dopamine (20 μM) for 1 h followed by penfluridol (7.5 μM) treatment for 24 h. Expression levels of CCAAT-enhancer-binding protein homologous protein (CHOP), LC3-I/II, and cleaved poly(ADP ribose) polymerase (PARP) were detected by a Western blot analysis. α-Tubulin was used as an equal loading control. **C** 786-O and A498 cells were pretreated with dopamine (20 μM) for 1 h followed by penfluridol (2.5 μM) treatment for 48 h. OCT4 mRNA levels were measured by an RT-qPCR. Quantitative results of OCT4 mRNA levels were adjusted to GAPDH mRNA levels. (**D** and **E**) ccRCC cells were treated with penfluridol at the indicated concentration with or without dopamine (20 μM) for 24 h, and the proliferative and colony-forming abilities were respectively determined by CCK-8 (**D**) and colony formation (**E**) assays. Values from **C**–**E** are presented as the mean ± standard deviation (SD) of three independent experiments. **p < 0.01 and ***p < 0.001 compared to the penfluridol treatment only group. **F** The cBioPortal database was used to analyze correlations between DRD2 and OCT4, Nanog, or GLI1 in patients with ccRCC. **G** Kaplan-Meier plot of overall survival of patients with ccRCC stratified by DRD2 expression levels. The ccRCC dataset was retrieved from TCGA.

The mechanism of penfluridol against schizophrenia is thought to be blockade of DRs, especially DRD2 [20]. Actually, significantly higher DRD2 transcripts were observed in ccRCC compared to normal tissues from the GSE15641 dataset of the GEO database (Fig. 7A). We also observed that knockdown of DRD2 in RCC significantly reduced the cell proliferation and colony-forming abilities (Supplementary Fig. 9A and B). Until now, there has been no evidence that the anticancer activity of penfluridol is due to DRD2 antagonism. Herein, we used dopamine to compete with penfluridol for binding to DRD2 and found that combination treatment of ccRCC cells with dopamine and penfluridol respectively reversed penfluridol-induced upregulation of CHOP, cleaved PARP, and LC3-II and inhibition of OCT4 mRNA expression in ccRCC cells harboring the WT or mutant *VHL* (Fig. 7B and C). Functionally, dopamine respectively reversed penfluridol-induced inhibition of cell proliferation (Fig. 7D) and colony formation (Fig. 7E) in various ccRCC cell lines. Because dopamine is not a DRD2-specific agonist, we further used the selective DRD2 agonist, quinpirole, to confirm our results and observed that penfluridol-induced inhibition of cell proliferation was also significantly reversed by quinpirole in 786-O cells (Supplementary Fig. 10). In contrast, depletion of DRD2 further enhanced penfluridol-triggered ER stress, autophagy, and cytotoxicity (Supplementary Fig. 9C and D). These results suggested that DRD2 is involved in the penfluridol-induced increase in ER stress, inhibition of stemness, and autophagy-mediated apoptosis in ccRCC cells. In patients with ccRCC from TCGA database, we observed that DRD2 expression was positively correlated with GLI1, OCT4, and Nanog expressions (Fig. 7F) and poor prognoses of patients (Fig. 7G). Taken together, DRD2 might be a critical target involved in the anticancer activities of penfluridol in ccRCC cells.

## Discussion

Interesting epidemiologic studies indicated reduced cancer risk was observed in patients with various neurological diseases such as schizophrenia and PD, for which dopaminergic drugs were used. Recently, DRD2 was reported to be upregulated in various cancers and tied to the stemness of solid and nonsolid tumors [35]. Therefore, DRD2 antagonists might be promising repurposed drugs for cancer treatment. Actually, DRD2 antagonists were reported to have anticancer efficacy in cell culture and animal models in several cancer types [20, 22, 23, 35]. Nevertheless, the anticancer effects of DRD2 antagonists and the precise mechanisms of DRD2 signaling and their relationships to RCC progression remain poorly understood.

Herein, we used the DRD2 antagonist, penfluridol, to identify the critical role of DRD2 in treating RCC, especially ccRCC, and explored the relevant mechanisms for its possible therapeutic application. First, we demonstrated that penfluridol significantly inhibited cell proliferation and colony formation in concentration-dependent manners in vitro and attenuated the tumorigenic ability in vivo. A mechanistic investigation revealed that penfluridol treatment induced the ER stress-mediated UPR signaling pathway, GRP78/PERK/eIF2α/CHOP axis, which led to the autophagy-mediated apoptotic cell death of ccRCC cells. These results are similar to previous studies which showed that penfluridol-induced ER stress led to autophagy and ultimately induced cell apoptosis in pancreatic cancer [36, 37]. However, in this study, we observed that blocking of autophagy by several autophagy inhibitors just partly reversed penfluridol-induced inhibition of cell growth, but almost reversed the penfluridol-induced PARP cleavage. In contrast, blocking of ER stress can dramatically rescue penfluridol-induced inhibition of cell growth, suggesting autophagy-mediated apoptotic cell death might be one of the causes to explain the cytotoxic effect of penfluridol in ccRCC cells. We speculate that other forms of cell death such as necrosis or ferroptosis might be triggered by penfluridol-induced ER stress and these issues should be further investigated in our future work.

Moreover, we used the DRD2 agonists, dopamine and quinpirole, to compete with penfluridol for DRD2 binding and observed that co-treatment with a DRD2 agonist and penfluridol significantly reversed penfluridol-induced upregulation of ER stress and inhibition of cell growth, suggesting that DRD2 is a critical target of penfluridol’s anticancer activities in ccRCC. In pancreatic cancer, DRD2 inhibition by pimozide resulted in anticancer activity by inducing cell death through activating the cyclic adenosine monophosphate (cAMP)/protein kinase A (PKA) pathway leading to higher calcium levels and elevated ER stress, thus inducing apoptosis [17]. In addition to pancreatic cancer, He et al. showed that EGF receptor (EGFR) overexpression was associated with poor in vitro and in vivo responses to DRD2 antagonists [38], suggesting the mechanisms by which DRD2 signaling contributes to cancer progression appeared to be cell-type dependent. Indeed, we also observed that DRD2 expression was positively and negatively correlated with PRKACA (PKA catalytic subunit) and EGFR expression in patients with ccRCC from TCGA database (Supplementary Fig. 11). The roles of cAMP/PKA and EGFR in the anticancer effects of penfluridol for ccRCC need to be further investigated in the future.

Recently, DRD2 was found in CSCs. For example, treatment with the DRD2 antagonist, thioridazine, revealed in vivo anti-CSC activity by reducing leukemic stem cell function in forming leukemia [39]. In our present study, results from sphere-formation and xenotransplantation limiting dilution assays also showed that penfluridol exhibits in vitro and in vivo anti-CSC activities in ccRCC. Gassenmaier et al. indicated that CXC chemokine receptor 4 (CXCR4)^+^ RCC cells presented CSC characteristics, such as increased resistance to TKIs, higher sphere-forming ability, and tumor growth-inducing potential in vivo, and expressed high levels of the stem cell-associated markers, OCT3/4, Nanog, and Sox2 [40]. Herein, we found that penfluridol transcriptionally inhibited OCT4 and Nanog expressions and enhanced the response to a TKI, sunitinib, in various ccRCC cells, but the role of CXCR4 in the anti-CSC activity of penfluridol needs to be further investigated.

GLI1 is one of three GLI transcription factors within the Hh pathway and was reported to modulate the CSC self-renewal ability through transcriptionally inducing upregulation of OCT4, Nanog, and Sox2 [32]. In addition to being a terminal effector, GLI1 also acts as a GLI target gene that elicits signal amplification [41]. Previous studies [42] and our present results indicated that GLI1 is upregulated in ccRCC compared to normal tissues and was correlated with the poor prognosis of patients with ccRCC. In addition, GLI1 overexpression by normal kidney tubular cells showed enhanced tumorigenic abilities with tumor-formation rates in an animal model [42], and the GLI1 expression level was significantly correlated with OCT4 and Nanog levels in ccRCC specimens, suggesting that GLI1 plays a critical role in the initiation and progression of ccRCC via upregulating OCT4 and Nanog. In vitro, we observed that penfluridol treatment suppressed GLI1 expression in ccRCC cells harboring WT or mutant *VHL*. The GLI1-specific inhibitor, GANT58, can further enhance the penfluridol-induced inhibition of proliferation, OCT4 and Nanog mRNA expressions, and induction of apoptosis. Regardless of the *VHL* status, GLI1 was suggested to be an important target for the growth-suppressive effect of penfluridol in ccRCC cells. Actually, Zhou et al. also indicated that GLI1 expression was not affected by the *VHL* status in ccRCC [42]. Recently, Ranjan et al. demonstrated that penfluridol suppresses tumor growth of GBM by inhibiting Akt-mediated GLI1 expression [32]. In ccRCC, Zhou et al. showed that the phosphatidylinositol 3-kinase (PI3K)/Akt pathway could potentiate the expression and activation of GLI1 in ccRCC cells [42], suggesting that penfluridol may suppress GLI1 expression through inhibiting the PI3K/Akt pathway in ccRCC.

**Fig. 8:**
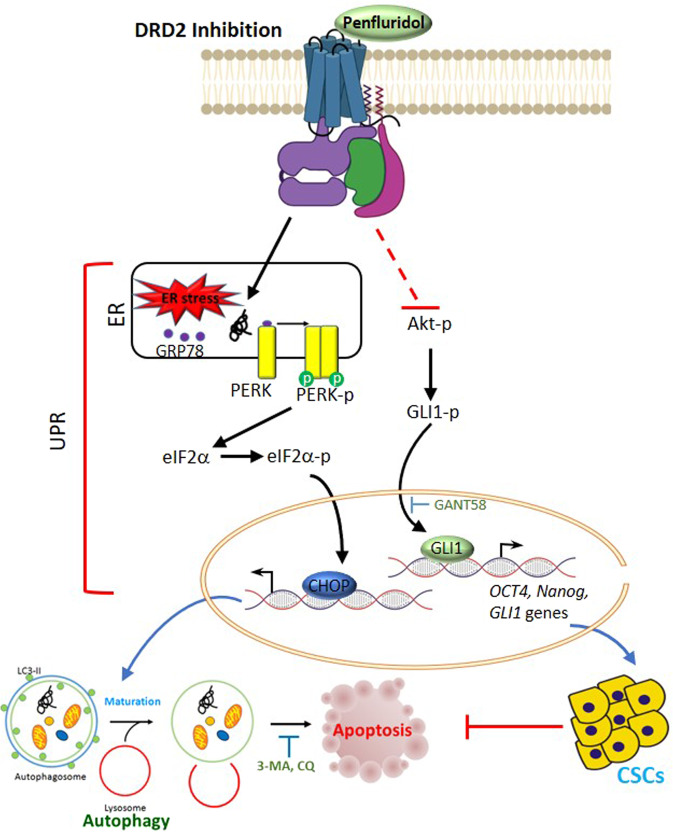
A working model shows the molecular mechanism underlying the ability of the dopamine receptor D2 (DRD2) antagonist penfluridol to retard growth of clear cell renal cell carcinoma (ccRCC) cells. The anticancer activity of penfluridol on ccRCC cells was attributed to induction of ER stress leading to UPR-mediated autophagy and subsequently induction of apoptosis. Meanwhile, penfluridol not only triggers apoptosis but also attenuates stemness of ccRCC cells via inhibiting Akt/GLI1/OCT4/Nanog axis. Bold dashed lines indicate hypothetical pathways which might be inhibited by penfluridol.

The present data demonstrated the novel anticancer mechanism of the DRD2 antagonist, penfluridol, in human ccRCC which harbors the WT or mutant *VHL*. Penfluridol can trigger the ER stress-elicited GRP78/PERK/eIF2α/CHOP signaling pathway of the UPR, and suppress the Akt/GLI1/OCT4/Nanog axis which respectively led to autophagy and stemness inhibition and further induced apoptosis of ccRCC cells; the mechanism is schematically illustrated in Fig. 8. Our present findings strongly support repurposing penfluridol as a drug for targeting DRD2 to treat ccRCC.

## Supplementary information

Supplementary data

Original Data File

Reproducibility checklist

## Data Availability

All the data in the current study are available from the corresponding authors upon reasonable request.
