# Supplementary material for: Targeting DRD2 by the antipsychotic drug, penfluridol, retards growth of renal cell carcinoma via inducing stemness inhibition and autophagy-mediated apoptosis
Source: Cell Death Dis. 2022 Apr 23;13(4):400. doi: 10.1038/s41419-022-04828-3 (PMC9035181; doi:10.1038/s41419-022-04828-3)
Supplement: Supplementary file 1 — Supplementary data [file 41419_2022_4828_MOESM1_ESM.pdf]

Supplementary data

Title:

Targeting DRD2 by the antipsychotic drug, penfluridol, retards growth of renal cell carcinoma via inducing stemness inhibition and autophagy-mediated apoptosis

Min-Che Tung†, Yung-Wei Lin†, Wei-Jiunn Lee, Yu-Ching Wen, Yu-Cheng Liu, Ji-Qing Chen, Michael Hsiao, Yi-Chieh Yang*, and Ming-Hsien Chien*

*Correspondence: Dr. Yi-Chieh Yang (E-mail: rafiyang@tmu.edu.tw) or Dr. Ming-Hsien Chien (E-mail: mhchien1976@gmail.com)

Supplementary Figures

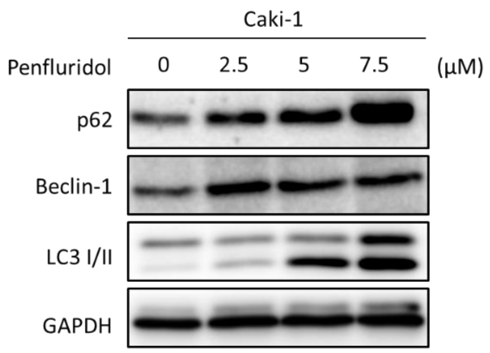

Supplementary Figure 1. Effects of different concentrations of penfluridol on autophagy induction in Caki-1 cells. The expression of p62 and Beclin-1 and turnover of LC3 levels were examined with a Western blot analysis after treating with penfluridol for 24 h at the indicated concentration. GAPDH was used as an equal loading control.

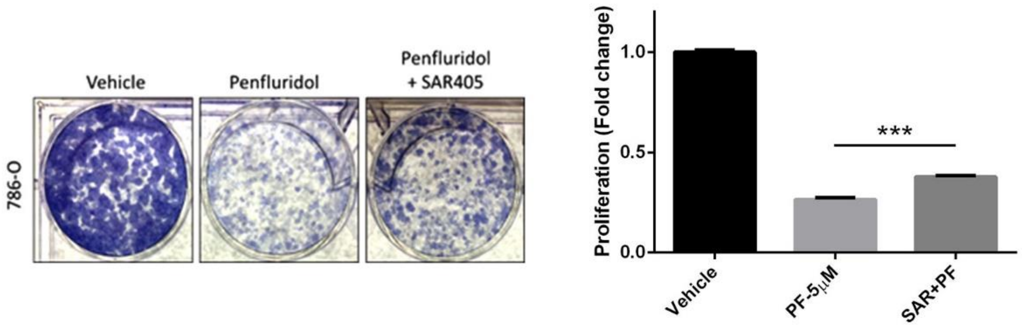

Supplementary Figure 2. Inhibition of VPS34 by SAR405 partly reverses the penfluridol-mediated suppression of colony formation in 786-O cells. Cells were pretreated with SAR405 (1 μM) for 1 h followed by penfluridol treatment for another 24 h. The death-inducing effects of penfluridol on cells were determined by CCK8 assay (right panel) and counting the colonies formed (left panel).

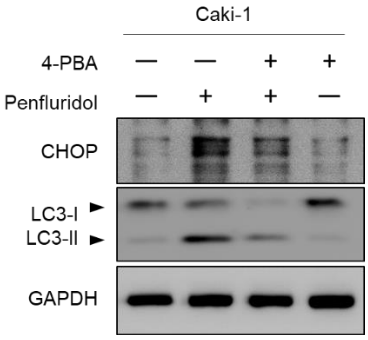

Supplementary Figure 3. Blockade of the ER stress reverses the penfluridol-induced autophagy in Caki-1 cells. Cells were pretreated with 1 mM 4-phenylbutyric acid (4-PBA)

for 1 h followed by penfluridol treatment for 24 h. The expressions of LC3 turnover and CHOP were examined by a Western blot analysis.

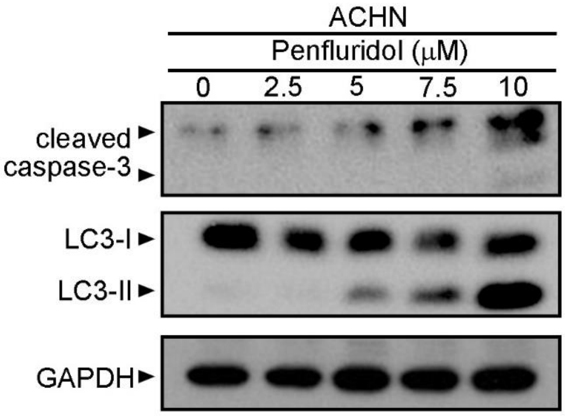

Supplementary Figure 4. Effect of penfluridol on apoptosis and autophagy induction in ACHN papillary renal cell carcinoma (pRCC) cells. LC3 turnover and caspase 3 cleavage were assessed by a Western blot analysis after treating ACHN cells with penfluridol for 24 h at the indicated concentrations. GAPDH was used as an equal loading control.

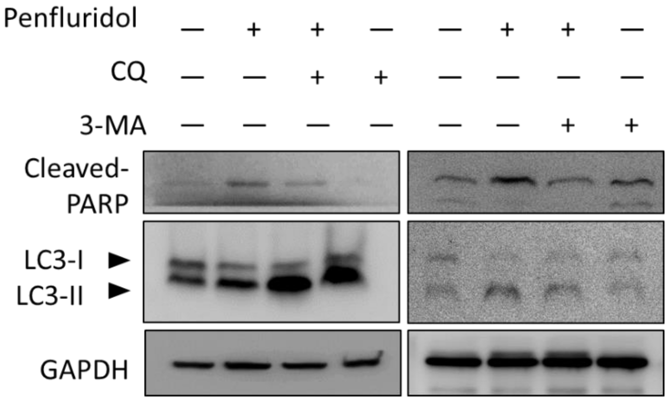

Supplementary Figure 5. Autophagy inhibition attenuates penfluridol-induced poly(ADP ribose) polymerase (PARP) cleavage in 786-O cells. Cells were pretreated with 20 μM of 3-methylamphetamine (3-MA) or chloroquine (CQ) for 1 h followed by penfluridol (7.5 μM) treatment for 24 h. Expression levels of LC3 and cleaved poly(ADP ribose) polymerase (PARP) were detected by a Western blot analysis, and GAPDH was used as an equal loading control.

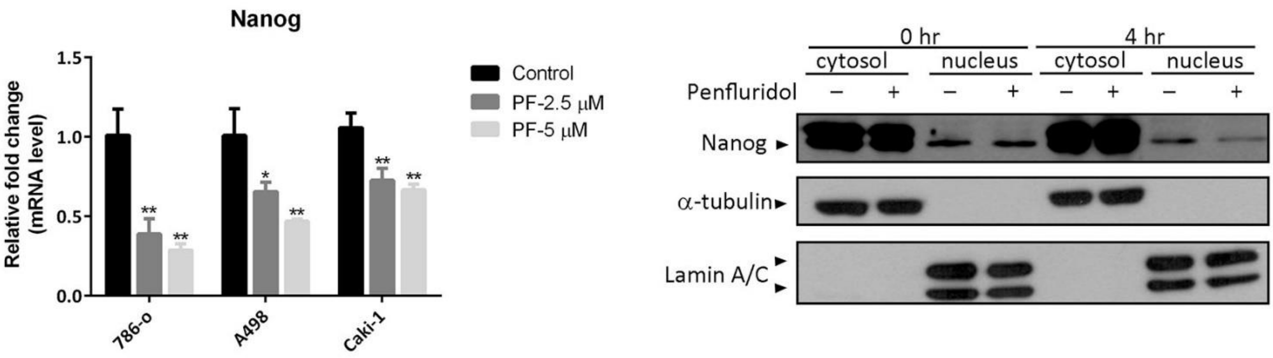

Supplementary Figure 6. Effect of penfluridol on the mRNA expression and protein nuclear localization of Nanog in clear cell renal cell carcinoma (ccRCC) cells. Left panel, A498, 786-O, and Caki-1 cells were treated with vehicle or penfluridol at the indicated concentrations for 72 h to detect mRNA levels of Nanog using an RT-qPCR. Quantitative results of Nanog mRNA levels were adjusted to GAPDH mRNA levels. Right panel, A498 cells were treated with 7.5 μM penfluridol or vehicle for 4 h. Cells were harvested and fractionated into cytoplasmic and nuclear fractions and then subjected to a Western blot analysis to detect Nanog levels. α-Tubulin and lamin A/C were respectively used as the cytosolic and nuclear loading controls.

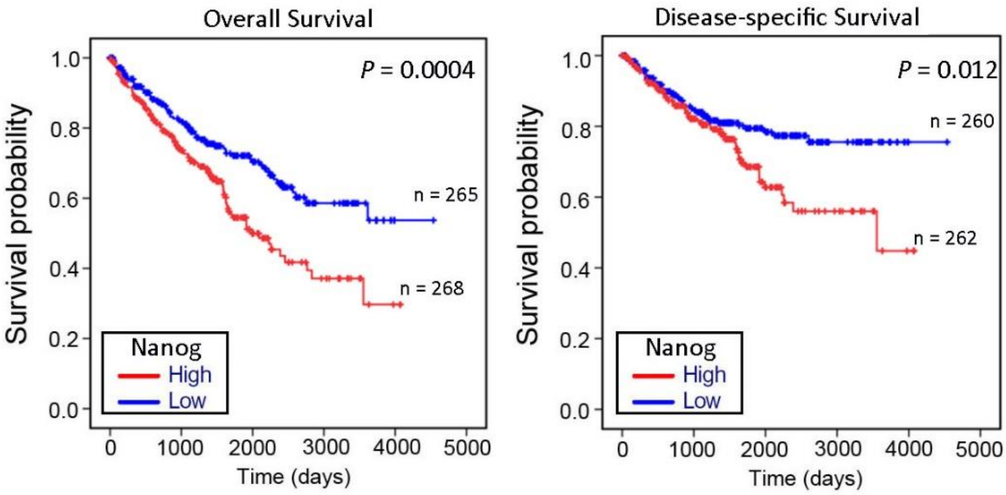

Supplementary Figure 7. Kaplan-Meier plots of overall (left panel) and disease-specific (right panel) survival of patients with clear cell renal cell carcinoma (ccRCC) stratified by Nanog expression levels. The *p* value indicates a comparison between patients with Nanog^{high} and Nanog^{low}. The ccRCC dataset was retrieved from TCGA.

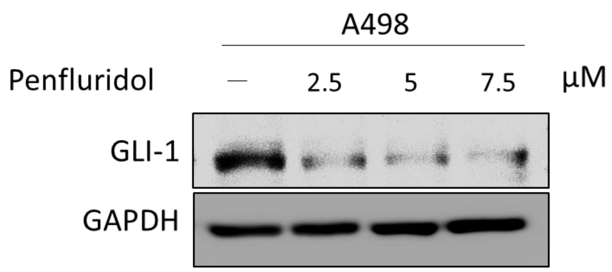

Supplementary Figure 8. Penfluridol reduces the GLI1 expression in A498 cells. Cells were received the indicated concentration of penfluridol for 24 h and the expression of GLI1 were examined by a Western blot analysis. GAPDH was used as an equal loading control.

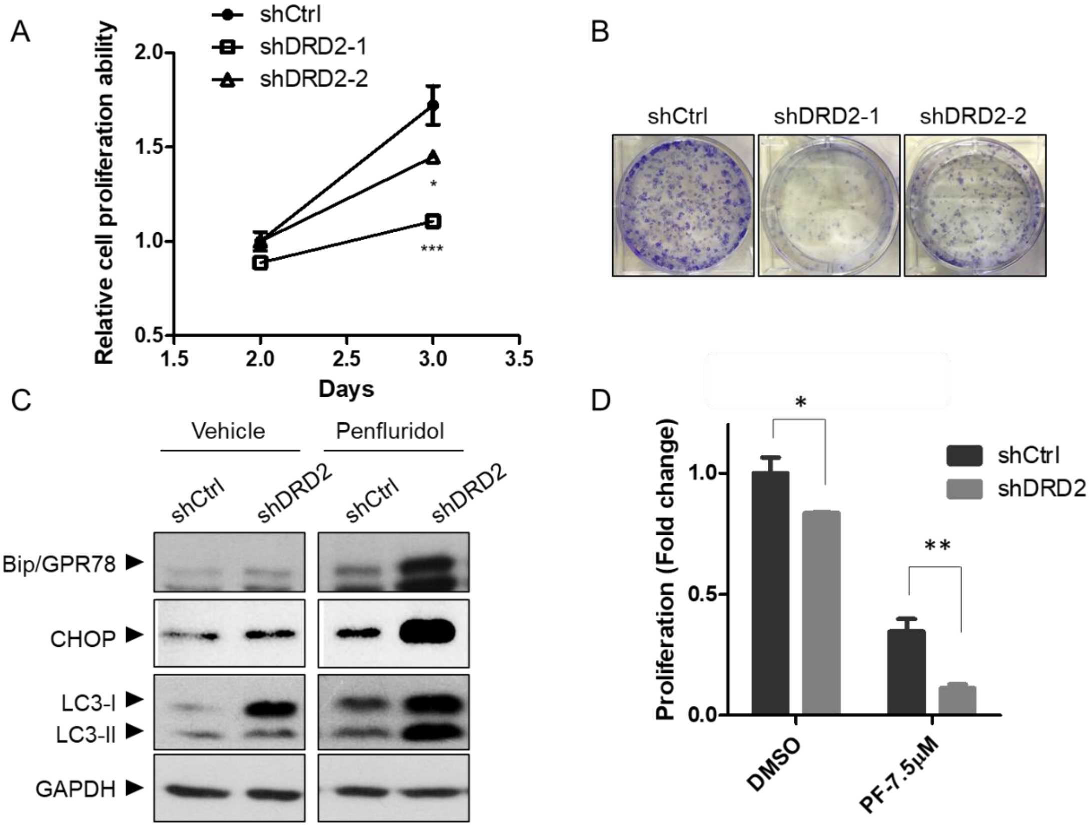

Supplementary Figure 9. Knocking down of DRD2 expression enhances the penfluridol-induced increase of ER stress and inhibition of cell proliferation. 786-O cells were infected with sh-control (shCtrl)/sh-DRD2 virus for 48 hours, and the cells were re-seeded to 6-cm or 96-well dishes for following experiments. (A) Proliferation rates of DRD2-knockdown 786-O cells were measured by performing the MTT assay. Proliferation rates were significantly decreased in shDRD2 stably transfected cells for

72 h. (B) Colony-forming abilities of 786-O after knocking down the DRD2 for 7 days. (C) Western blot analysis of GRP78, CHOP, and LC3 expressions in cells after transducing shDRD2 or shCtrl and treatment with penfluridol (7.5 μM) or the vehicle. (D) shDRD2 stably transfected cells were treated with penfluridol or the vehicle for 48 h, and the proliferative ability was analyzed by the MTT assays. Values are presented as the mean ± standard deviation (SD). * $p<0.05$, ** $p<0.01$, and *** $p<0.001$ compared to the shCtrl group.

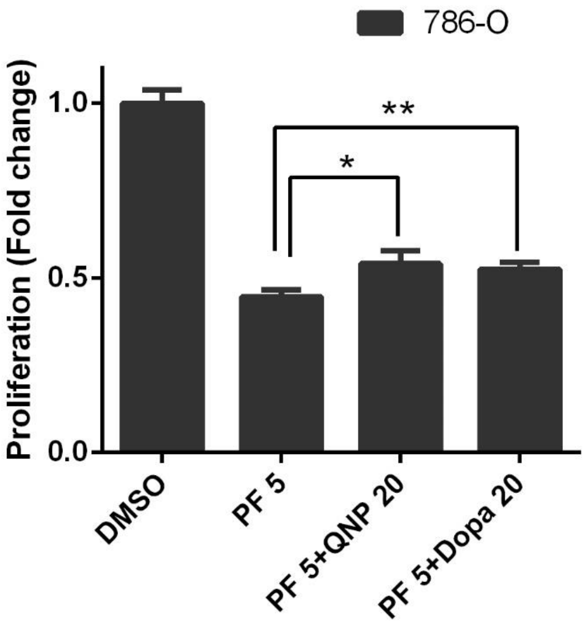

Supplementary Figure 10. The dopamine receptor D2 (DRD2)-specific agonist, quinpirole, significantly reverses penfluridol-induced inhibition of proliferation of clear cell renal cell carcinoma (ccRCC) cells. 786-O ccRCC cells were treated with penfluridol (5 µM) with or without quinpirole or dopamine (20 µM) for 24 h. The proliferative ability of cells was determined by a CCK-8 assay and values are presented as the mean ± standard deviation (SD) of three independent experiments. * $p < 0.05$ and ** $p < 0.01$ compared to the penfluridol treatment only group.

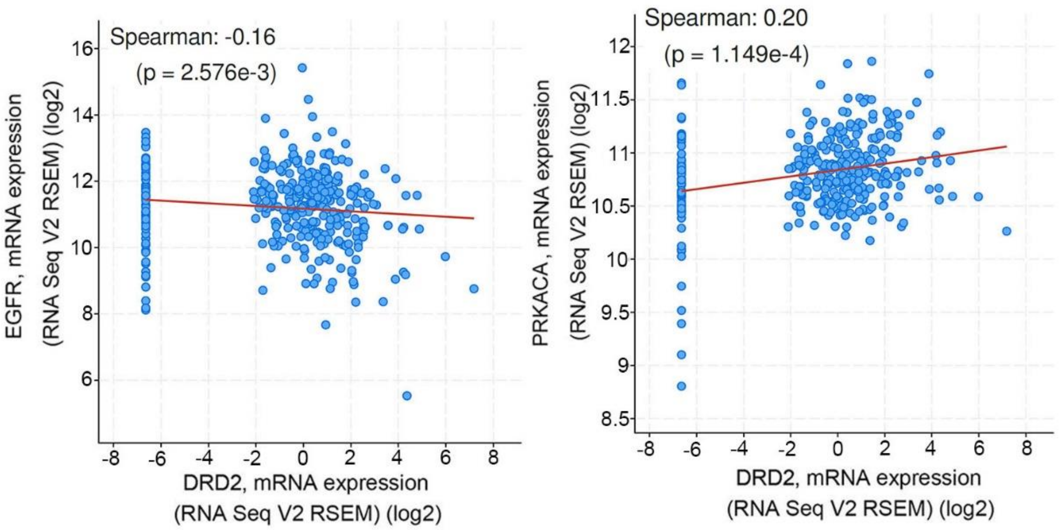

Supplementary Figure 11. Correlation analysis of TCGA clear cell renal cell carcinoma (ccRCC) database (TCGA, PanCancer Atlas) using the cBioPortal showed correlations of dopamine receptor D2 (DRD2) and protein kinase A (PKA; PRKACA) with epidermal growth factor receptor (EGFR) mRNA levels.
