# Supplementary material for: Targeting DRD2 by the antipsychotic drug, penfluridol, retards growth of renal cell carcinoma via inducing stemness inhibition and autophagy-mediated apoptosis
Source: Cell Death Dis. 2022 Apr 23;13(4):400. doi: 10.1038/s41419-022-04828-3 (PMC9035181; doi:10.1038/s41419-022-04828-3)
Supplement: Supplementary file 2 — Original Data File [file 41419_2022_4828_MOESM2_ESM.pdf]

Fig 2B

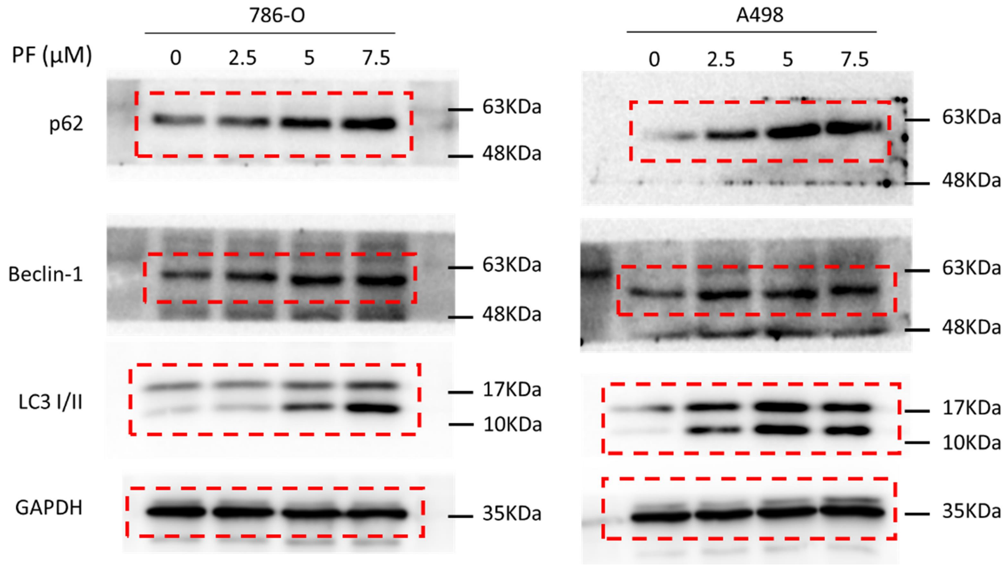

Fig 2C

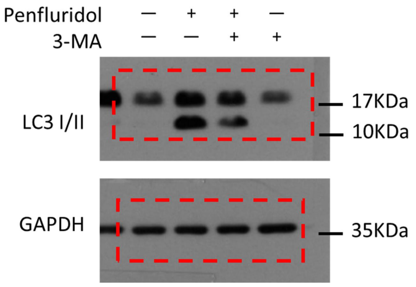

Fig 2E

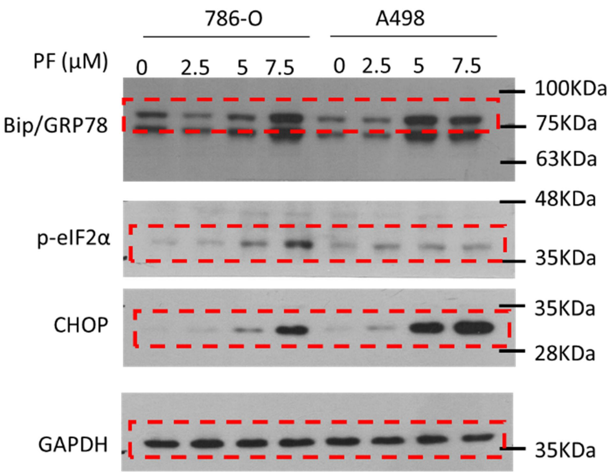

Fig 2F

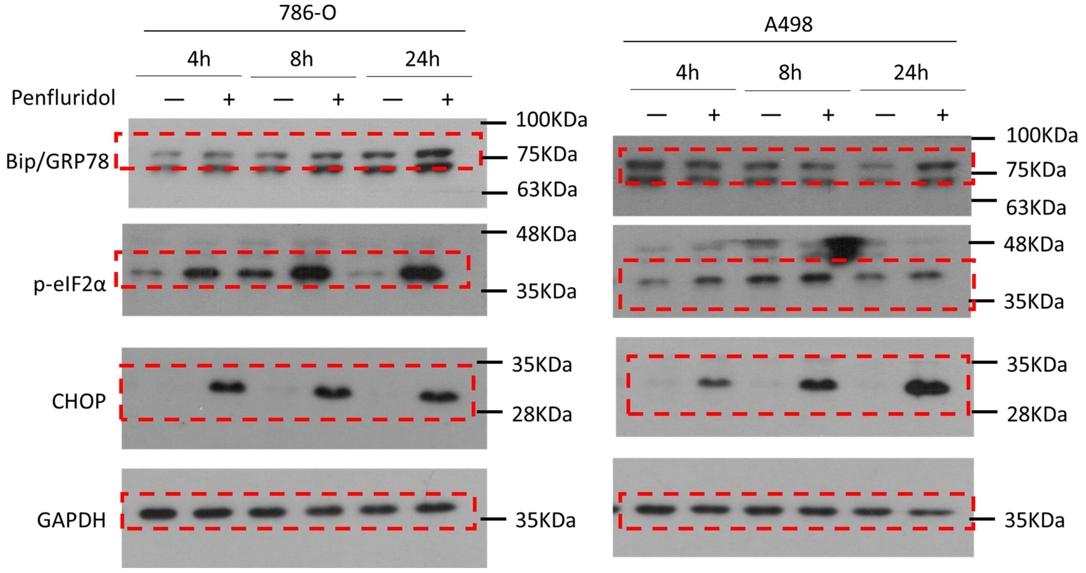

Fig 2G

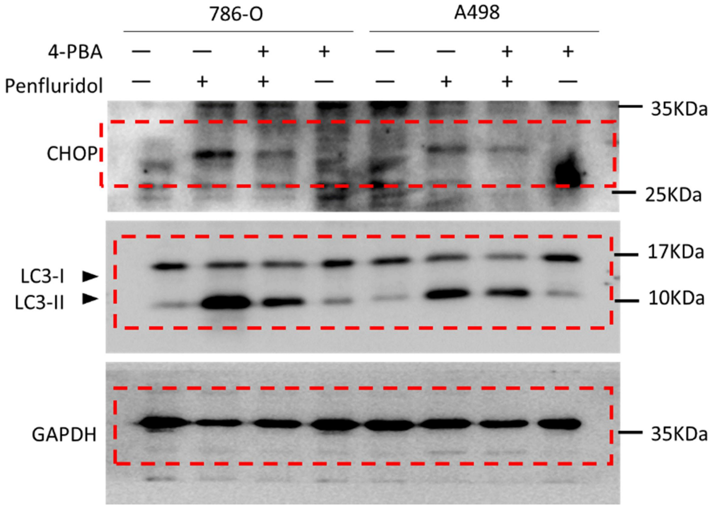

Fig 3A

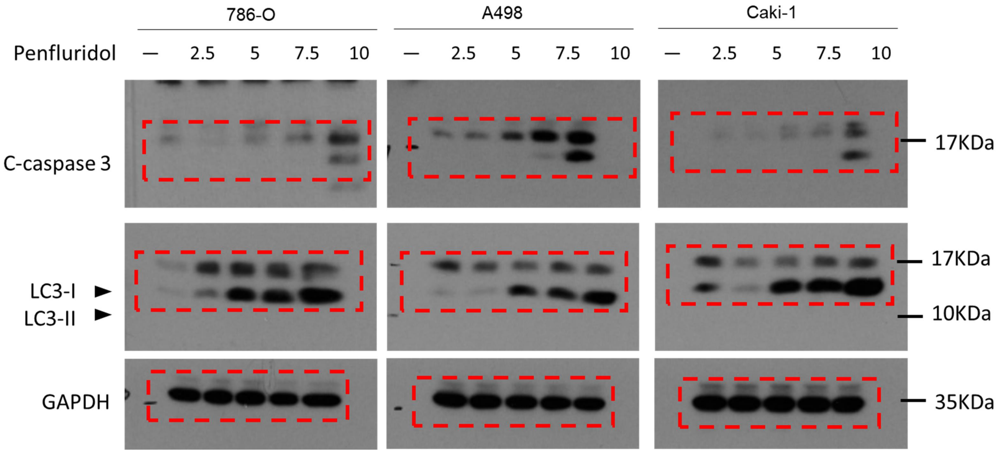

Fig 3C

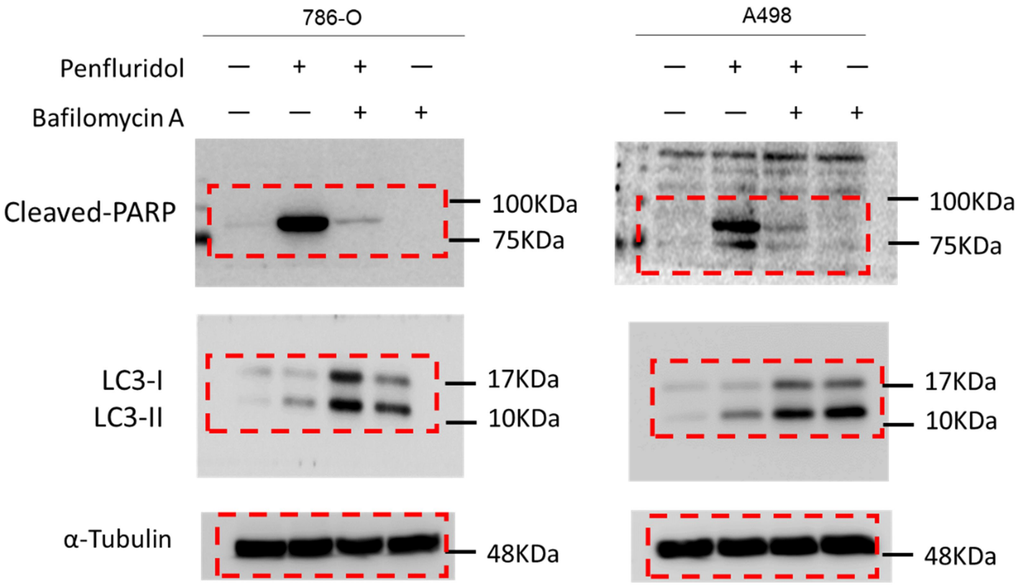

Fig 3D

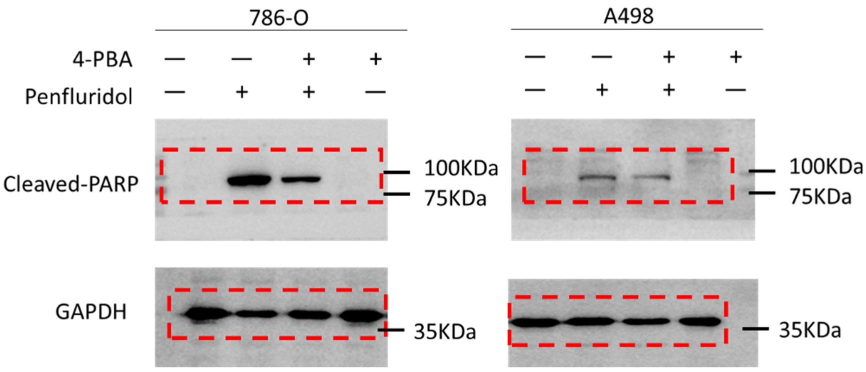

Fig 4D

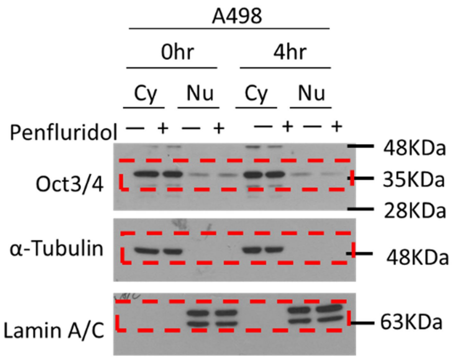

Fig 5A

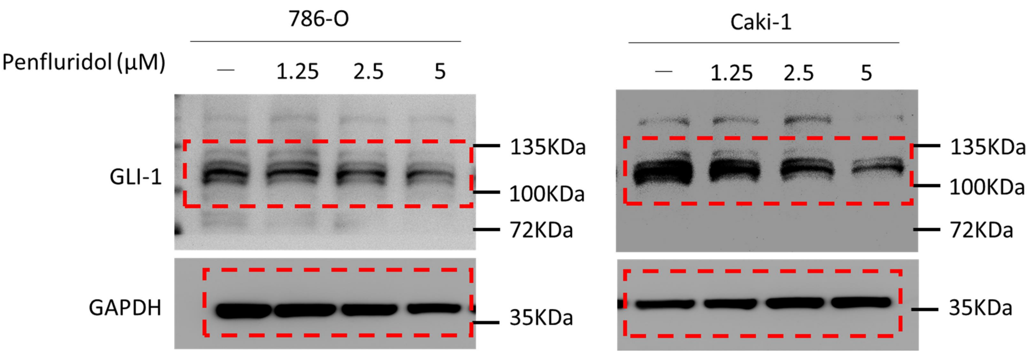

Fig 5D

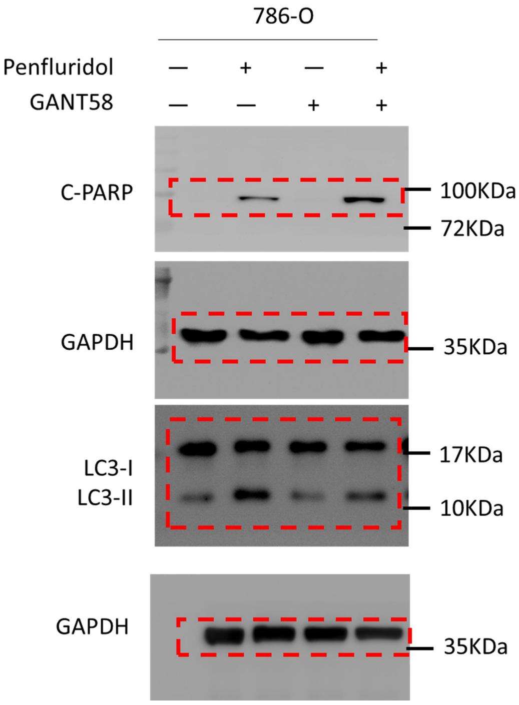

Fig 7B

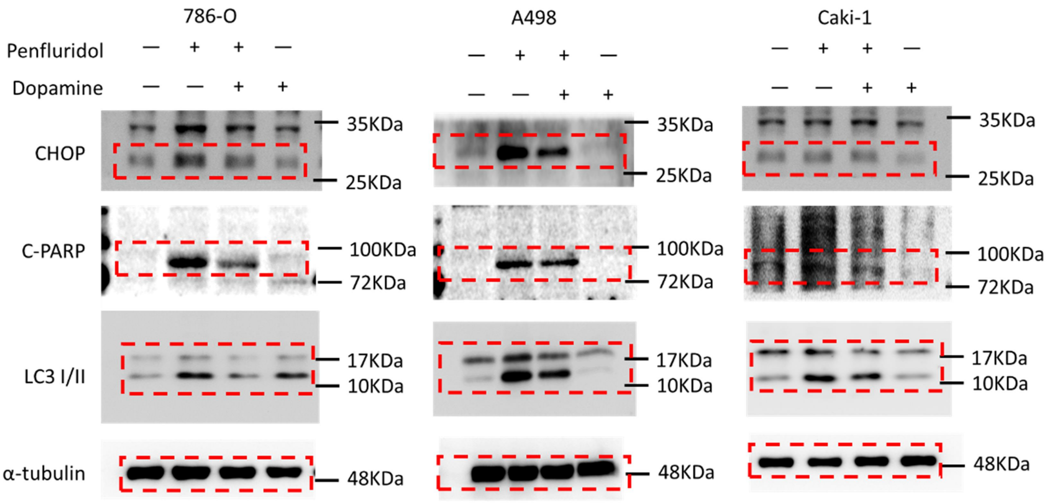

Sup Fig 1

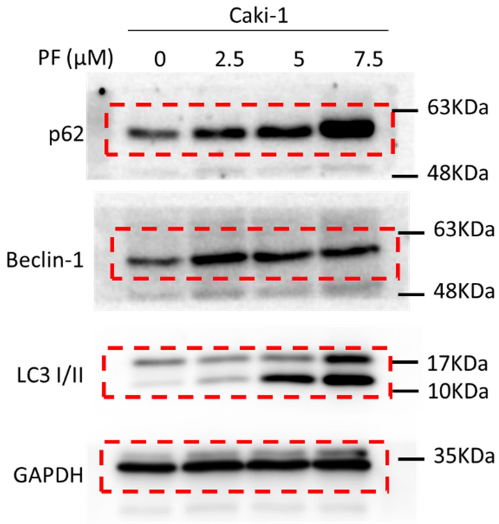

Sup Fig 3

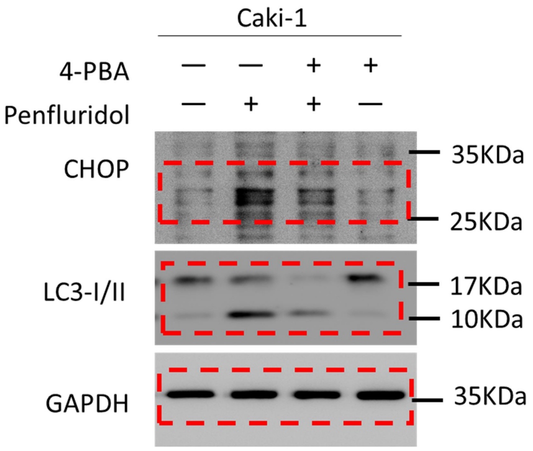

Sup Fig 4

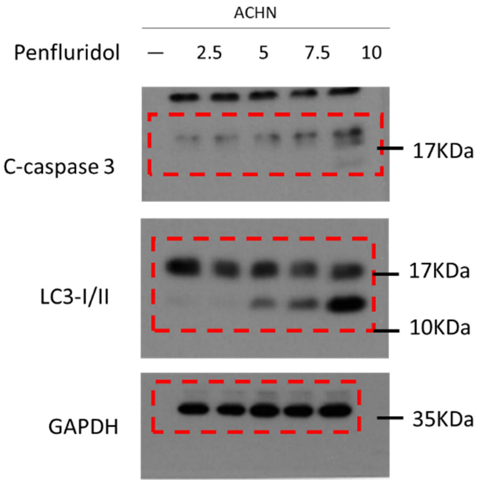

Sup Fig 5

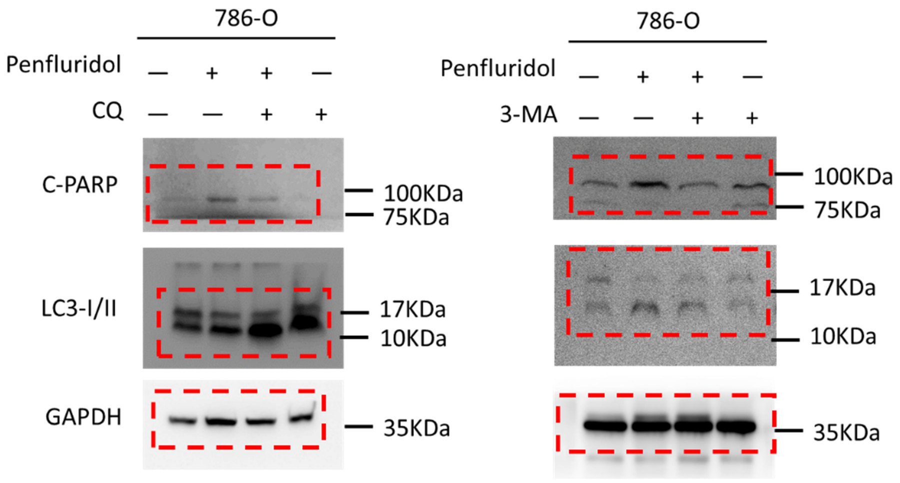

Sup Fig 6

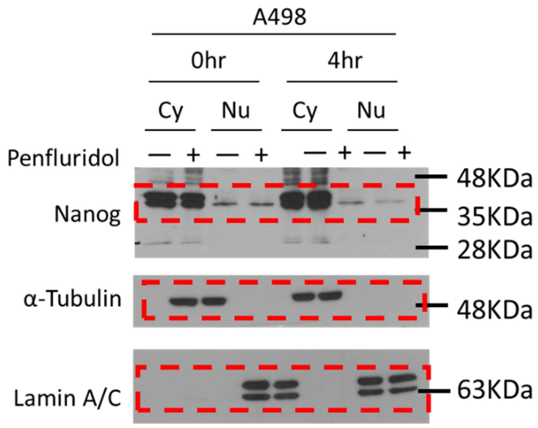

Sup Fig 8

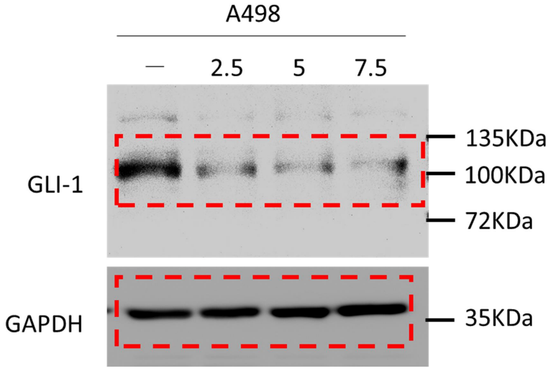

Sup Fig 9C

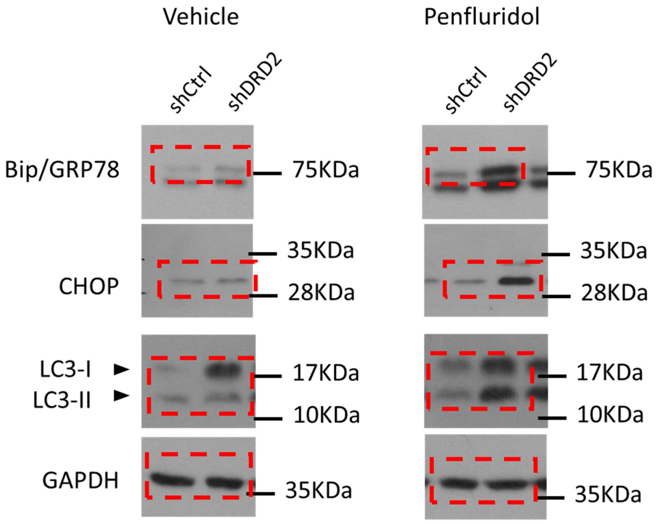

Vehicle
Penfluridol
shCtrl
shDRD2
shCtrl
shDRD2
Bip/GRP78
75KDa
75KDa
CHOP
35KDa
28KDa
35KDa
28KDa
LC3-I
LC3-II
17KDa
10KDa
17KDa
10KDa
GAPDH
35KDa
35KDa
